# Efficacy and Safety of Mobile App–Based Metamemory Cognitive Training for Mild Cognitive Impairment: Multicenter Randomized Clinical Trial

**DOI:** 10.2196/73464

**Published:** 2026-01-19

**Authors:** Sunyoung Kang, Jung-In Lim, Lukas Stenzel, Keun You Kim, Eosu Kim, Hong Jun Jeon, Doo-Heum Park, Hyun Kook Lim, YongSoo Shim, Jae-Won Jang, Yeshin Kim, Sanghoon Lee, Kee Hyung Park

**Affiliations:** 1 Department of Psychiatry & Institute of Behavioral Science in Medicine Yonsei University College of Medicine Seoul Republic of Korea; 2 Interdisciplinary Program in Cognitive Science Seoul National University Seoul Republic of Korea; 3 Cogthera GmbH Munich Germany; 4 Graduate School of Medical Science, Brain Korea 21 Project Yonsei University College of Medicine Seoul Republic of Korea; 5 Metabolism-Dementia Research Institute Yonsei University College of Medicine Seoul Republic of Korea; 6 Department of Psychiatry Konkuk University Medical Center Seoul Republic of Korea; 7 Department of Psychiatry Yeouido St. Mary's Hospital, College of Medicine Catholic University of Korea Seoul Republic of Korea; 8 Catholic Medical Center Institute for Basic Medical Science The Catholic University of Korea Catholic Medical Center Seoul Republic of Korea; 9 Department of Neurology St. Vincent's Hospital, College of Medicine Catholic University of Korea Seoul Republic of Korea; 10 Department of Neurology Kangwon National University Hospital, College of Medicine Kangwon National University Chuncheon, Gangwon-do Republic of Korea; 11 Emocog Inc. Seoul Republic of Korea; 12 Department of Neurology, College of Medicine Gachon University Gil Medical Center Incheon Republic of Korea

**Keywords:** cognitive decline, cognitive training, digital therapeutics, mild cognitive impairment, mobile app–based cognitive training

## Abstract

**Background:**

Metamemory training (MMT) offers a potential nonpharmacological approach to enhance cognitive function in individuals with mild cognitive impairment (MCI). While digital cognitive training improves accessibility, the effectiveness of mobile app–based MMT has not been evaluated in a randomized clinical trial.

**Objective:**

We aimed to evaluate the efficacy and safety of a mobile app–based MMT program, ET-101 (Cogthera), compared to a sham device control group in individuals with MCI.

**Methods:**

This multicenter, randomized controlled trial enrolled participants with MCI, recruited from 7 medical centers, and randomly assigned them to the ET-101 or control group (1:1 ratio). The intervention lasted 12 weeks, with a 12-week follow-up. The ET-101 group received metamemory-based multimemory strategy training and real-time feedback. Assessments of cognition, the daily activities of living, and the quality of life were conducted at baseline, week 12, and week 24. The primary outcome was the proportion of participants who showed cognitive improvement as assessed by the Alzheimer’s Disease Assessment Scale-Cognitive Subscale (ADAS-Cog)-14 at weeks 12 and 24. Secondary outcomes included changes in the scores of scales assessing cognition, daily activities, and quality of life. Safety analysis assessed adverse events and their relation to digital therapeutics.

**Results:**

In the full analysis set, 49 participants were included in the ET-101 group and 50 in the control group. At week 24, the proportion of responders who maintained or improved their ADAS-Cog-14 scores was significantly higher in the ET-101 group than in the control group (*P=*.002). Additionally, the ET-101 group showed a significant improvement in ADAS-Cog-14 scores at week 24 compared to baseline levels (estimates=–2.53; t_265_=–3.05; Bonferroni-adjusted *P*=.003). A subdomain analysis revealed significant improvements in the memory (estimates=–2.50; t_264_=–4.03; Bonferroni-adjusted *P*<.001) and language (estimates=–0.807; t_290_=–3.68; Bonferroni-adjusted *P*<.001) domains at week 24 in the ET-101 group compared to the control group. In the safety analysis, 6 adverse events occurred in the ET-101 group and 4 in the control group, but none were related to the interventions. The attrition rate in the ET-101 group was 22.4% (11/49).

**Conclusions:**

ET-101 significantly improved cognitive function compared to the sham device, with effects observed not only in the memory domain but also in the language domain, indicating a transfer effect. Therefore, ET-101 has the potential to provide effective MMT to a broader population with MCI by overcoming location and personnel limitations through a mobile app–based platform.

**Trial Registration:**

ClinicalTrials.gov NCT05938426; https://clinicaltrials.gov/study/NCT05938426

## Introduction

### Background

With the global population aging rapidly, the number of people affected by dementia is estimated to reach 153 million by 2050 [1]. Increasing evidence shows that timely diagnosis and treatment can slow the rate of cognitive deterioration, significantly reducing the burden on caregivers and social care systems [1]. As a result, effective interventions for mild cognitive impairment (MCI), which is at high risk of progressing, have become a priority in geriatric care [2].

Notably, anti–amyloid monoclonal antibody treatments, which have recently attracted attention in the management of MCI, may be appropriate only for patients who meet several eligibility criteria. Moreover, this treatment is associated with adverse effects that could limit continued use in some cases [3]. In contrast, cognitive training represents a relatively safe intervention with greater generalizability and applicability in the treatment of MCI, and it can also be used in combination with pharmacotherapy or as a stand-alone treatment option for patients who are unable to undergo or continue pharmacological therapy. According to a meta-analysis, cognitive training significantly improves memory function [4]. Moreover, magnetic resonance imaging–based studies have demonstrated that cognitive training improves cerebral blood flow and brain network connectivity, confirming the biological effects of cognitive interventions [5]. However, while cognitive training, as used in previous studies, improved older adults’ performance on practiced tasks, the studies did not consider the participants’ ability to self-perceive and regulate cognitive strategies during cognitively demanding tasks [6]. In addition, previous studies indicate that one of the key challenges in cognitive training for MCI is the lack of sustained effects once the training period ends [7,8]. These studies address the need for future interventions that aim to address and overcome this limitation and thereby enhance the real-life applicability of the learned strategies [7,8].

To address the limitations of previous methods of cognitive training, Flavell’s concept of “metamemory” can be beneficial [9]. Metamemory refers to one’s awareness of one’s own memory, including the contents and processes of their memory system [6,9]. Metamemory training (MMT) takes into account the concepts of meta-knowledge, meta-monitoring, and meta-judgement [10]. Meta-knowledge addresses how aging affects memory abilities, enabling older adults to learn strategies to deal with age-related cognitive decline [10]. In the meta-monitoring and meta-judgment components, older adults assess their own performance, enabling them to regulate their memorization ability by independently adjusting effective memory strategies according to the demands of the task at hand [10]. According to previous studies, MMT not only shows positive effects on everyday memory performance in older populations but also improves global cognitive functions while significantly reducing the frequency of memory-related discomfort in daily life [11-14].

Conventionally, the main limitation of cognitive interventions for MCI is their reliance on face-to-face interactions with professionals, which hampers the accessibility and continuity of training [15]. However, digitalization is ideal for facilitating the migration of care from clinics to patients’ homes, thereby overcoming constraints related to time and space. According to previous studies that evaluated the feasibility of digital cognitive therapy for MCI, patients adhered more to digital therapy compared to paper-and-pencil training. Additionally, in terms of effectiveness, a review of studies on computerized cognitive training for MCI found that its effect on cognitive function was not inferior to that of conventional cognitive interventions [15].

### Objectives

Thus, metamemory-based digital cognitive training can be expected to enhance the benefits of cognitive training in individuals with MCI by addressing the limitations of previous cognitive interventions. Based on this concept, ET-101 (Cogthera)—a mobile app–based MMT program—was developed. In this randomized clinical trial, we aimed to evaluate the efficacy and safety of ET-101 in older individuals with MCI compared to a control group using a sham device. To evaluate both the immediate and sustained effects of ET-101, we assessed its efficacy compared to sham controls at 2 time points that is, at 12 weeks, immediately after completion of the intervention, and 24 weeks, corresponding to a 12-week follow-up after the end of the intervention. This study aimed to evaluate the following hypotheses: first, for the primary outcome, we compared responder proportions (defined as participants showing maintenance or improvement in Alzheimer’s Disease Assessment Scale-cognitive Cognitive Subscale [ADAS-Cog]-14 scores) between the ET-101 and sham control groups, hypothesizing that the responder rate would be higher in the ET-101 group. Second, regarding secondary outcomes, we assessed changes from baseline to 12- and 24-week follow-ups in validated measures of cognitive function, activities of daily living (ADL), and quality of life, hypothesizing that greater improvements would be observed in the ET-101 group versus the sham control group. Finally, exploratory analyses were conducted to determine whether improvements on ADAS-Cog-14 were predominantly observed in the memory, language, or praxis domains to identify which specific cognitive functions were most responsive to ET-101.

## Methods

### Study Design and Participants

This was a multicenter, 24-week parallel, randomized, sham device–controlled study of ET-101. The participants were recruited through memory clinics of 7 medical centers in the Republic of Korea. The participants were recruited from June to December 2023. Participants who met the following inclusion criteria were included in the study: (1) individuals aged 55-85 years; (2) patients diagnosed with amnestic MCI by trained psychiatrists or neurologists according to Petersen’s criteria, characterized by cognitive complaints and objective memory impairment determined by either the Consortium to Establish a Registry for Alzheimer’s Disease Neuropsychological Assessment Battery (CERAD-NP) or the Seoul Neuropsychological Screening Battery (SNSB) verbal learning test [16,17]. Memory impairment was defined as a *z* score of –1 or lower on at least one of the following subtests: an immediate word recall test, a delayed word recall test, or a delayed word recognition part of the verbal learning test in CERAD-NP or SNSB, while maintaining essentially normal functional activities; (3) a Mini Mental State Examination (MMSE) score of 27 or lower [18]; (4) not meeting the diagnostic criteria for dementia; (5) a global Clinical Dementia Rating score of 0.5 [19]; (6) taking a stable dose for at least 12 weeks prior to randomization if prescribed acetylcholinesterase inhibitors or memantine; (7) having a study partner who spends more than 8 hours per week with the participant and agrees to assist with the participant’s follow-up and clinical evaluations; (8) possession of a personal smartphone and no difficulties using mobile apps; (9) the ability to make phone calls to a study partner independently using the smartphone; (10) no difficulties reading or writing in Korean; and (11) adequate vision and hearing for participating in the clinical trial.

Participants were excluded from the study if any of the following exclusion criteria were met: (1) a history of transient ischemic attack, stroke, or seizure within the past 12 months; (2) a history of severe psychiatric disorders or currently showing unstable psychiatric symptoms; (3) active suicidal intent as assessed by the Columbia-Suicide Severity Rating Scale or a history of treatment for suicidal behavior within the past 5 years [20]; (4) unstable findings upon a physical examination, neurological examination, in vital signs, or the presence of an ongoing unstable physical illness; (5) substance dependence within the past 2 years; (6) the use of prohibited medications as outlined in Table S1 in Multimedia Appendix 1; (7) scheduled to undergo surgery requiring general anesthesia; or (8) participation in any form of cognitive intervention within the past 3 months. Neurologic and psychiatric history, including past substance dependency, current symptoms, physical status, and neurological function, was assessed through clinical interviews conducted by trained psychiatrists and neurologists.

A statistician, independent of the clinical team, carried out the block randomization task, assigning participants to either the study group or the control group at a 1:1 ratio. Throughout the trial, psychiatrists, neurologists, and outcome raters were blinded to the treatment assignment. 

The intervention and control groups adhered to an identical visit schedule. Participants completed 5 visits (0-4): screening (visit 0) included screening for study eligibility; week 0 (visit 1, baseline) included baseline demographic data collection and clinical assessments of cognition, function, and quality of life, along with randomization; week 6 (visit 2) investigated compliance with the study protocol and the occurrence of adverse events (AEs); week 12 (visit 3, at the end of the 12-week treatment period) reassessed the same variables from visit 1 and visit 2; and week 24 (visit 4, follow-up assessment 12 weeks after treatment completion) reassessed the same variables from visit 3.

### Sample Size Calculation

Sample size was calculated based on a 2-sample proportion test. Delayed recall has been shown to have the highest predictive accuracy (95.2%) in differentiating dementia from MCI [21]. In a previous study, the proportion of participants showing an improvement on delayed recall was 82.8% in the intervention group and 45.16% in the control group [12]. With a 2-sided α of .05 and power of 0.90, and assuming a 1:1 allocation ratio, the minimum sample size per group was determined to be 32 participants. Anticipating a dropout rate of 35%, we intended to enroll 50 participants per group.

### Intervention

#### Study Group: ET-101

Study group participants used ET-101, a mobile app–based MMT program incorporating multimemory strategies. ET-101 is based on an MMT program developed by Youn and colleagues [22] and was produced by Emocog Inc. We previously introduced offline and smart speaker–based versions of this MMT program, and their effectiveness has been well validated [12,14].

Real-time metamemory teaching is a primary feature of ET-101. The training agent of ET-101 interacts with users through voice-based, 2-way communication to enhance metamemory, including meta-knowledge, meta-monitoring, and meta-judgment. Regarding meta-knowledge, on the first day, the agent begins the training by asking users questions about their long-term memory encoding and the memory strategies they currently use in daily life. Additionally, during the training sessions, the agent introduces multimemory strategies that can enhance long-term memory and explains how these strategies can improve memory performance. This helps users gain objective insight into their cognitive levels and understand how to compensate for cognitive deficits. Regarding meta-monitoring and meta-judgment, the agent explains the daily trends in users’ memory spans based on their training results. The agent identifies users’ strong and weak memory strategies, helping them recognize their cognitive strengths and weaknesses. Additionally, at the end of the training, the agent provides guidance on how to apply the learned strategies to daily life, facilitating the generalization of memory strategy use. Throughout the metamemory process, the agent provides personalized feedback, adjusts difficulty levels, and modifies training tasks based on users’ responses and training results.

ET-101 includes cognitive exercises specifically designed to incorporate memory strategies focusing on attention, imagination, and associations. According to the memory formation process and neuroscience findings, these strategies have been shown to effectively address issues with memory encoding and retrieval commonly observed in older people with cognitive decline [23,24]. Furthermore, attention, imagination, and association processes are known to strengthen related brain regions, including the prefrontal cortex, hippocampus, parahippocampus, precuneus, and the cingulum that connects these areas [23,25]. Attention training includes exercises that enhance trainees’ selective focus on stimuli to be memorized, as well as tasks aimed at improving the processing speed and increasing working memory. Imagination training facilitates the transition to long-term memory by linking spatial-temporal memory and visual imagery associated with the objects to be memorized. In association training, 2 or more pieces of information that need to be remembered are connected and assigned mutual meaning, thereby strengthening the semantic cue to aid in long-term memory formation. The detailed content of each activity can be found in Table S2 in Multimedia Appendix 1, and example screenshots are available in Multimedia Appendix 2.

The study group engaged in a 12-week training program using ET-101. The program was structured to involve 2 sessions each day, 7 days a week, lasting approximately 15 minutes per session. The initial session includes 3 representative cognitive exercises, with 1 session each for attention, imagination, and association. In the later session, 4 additional exercises are conducted, selected from 9 available options, excluding the 3 exercises from the initial session, based on a personalized algorithm (Table S3 in Multimedia Appendix 1). A push alarm was provided daily to encourage training.

#### Control Group: Sham Device

The control group used a sham device over a 12-week period, which featured cognitive assessments and gamification elements but did not include any cognitive training. In the sham device, on the first day, users are asked the same questions as in ET-101 about their long-term memory encoding and the memory strategies they currently use in daily life, presented in a conversational interaction format with the agent. From the second day onward, it offered a gamification element to encourage daily log-ins, in this case displaying a flower that gradually grows. No content related to cognitive training was provided. The guidelines for program usage frequency were identical to those followed by the study group.

### Measures

The efficacy of digital therapeutics was evaluated in terms of cognition, ADL, and quality of life. The scales listed below were assessed by a trained psychologist in a face-to-face manner.

#### Alzheimer’s Disease Assessment Scale-Cognitive Subscale-14

ADAS-Cog-14 was used to assess the severity of cognitive dysfunction [26]. ADAS-Cog-14 consists of the following 14 tasks: (1) word recall, (2) commands, (3) constructional praxis, (4) delayed word recall, (5) naming, (6) ideational praxis, (7) orientation, (8) word recognition, (9) maze, (10) number cancellation, (11) remembering instructions, (12) comprehension, (13) word-finding difficulty, and (14) spoken language ability. The total ADAS-Cog-14 score ranges from 0 to 90 points, calculated by summing the number of errors made on each task. A score of 0 indicates no impairment, while a score of 90 reflects the maximum level of impairment. ADAS-Cog subdomains were grouped into 3 domains: memory, language, and praxis [27]. The composite score range for each subdomain is as follows: memory (0-40), language (0-25), and praxis (0-15), with lower scores indicating less impairment.

#### Korean Mini Mental State Examination, 2nd Edition

MMSE is a brief neuropsychological test that provides an overview of the cognitive function in individuals with cognitive decline [28]. In this study, we used the latest revised version, the Korean Mini Mental State Examination, 2nd Edition (K-MMSE-II), whose validity has already been established [18]. It tests 5 areas of cognitive function: orientation, registration, attention and calculation, recall, and language. The scale ranges from 0 to 30 points, where higher scores indicate less impairment.

#### Clinical Dementia Rating-Sum of Boxes

Clinical Dementia Rating-Sum of Boxes (CDR-SB) is used to assess the stage of severity in Alzheimer dementia or MCI [29]. The score is determined through interviews with patients and informants, evaluating cognitive functioning across 6 domains: memory, orientation, judgment and problem-solving, community affairs, home and hobbies, and personal care. Each domain is scored from 0 to 3, and the CDR-SB outcome is calculated by summing the scores across all domains, resulting in a total score ranging from 0 to 18. Higher scores indicate greater impairment.

#### Alzheimer’s Disease Composite Score

Alzheimer’s Disease Composite Score (ADCOMS) is a tool developed to measure clinical progression and treatment effects in patients with cognitive decline [30]. ADCOMS comprises a total of 12 items, specifically 4 items from ADAS-Cog, 2 items from the MMSE, and all 6 items from CDR-SB. The total score is calculated by applying predetermined weights to each item. Total ADCOMS values range from 0 to 1.97, with higher scores indicating greater impairment.

#### Digit Symbol Coding

Digit Symbol Coding (DSC) is designed to assess frontal and executive functions [16]. Participants are shown symbols corresponding to numbers from 1 to 9 and are asked to draw the appropriate symbol in 133 blank spaces. Each correctly drawn symbol is awarded 1 point, resulting in a total score ranging from 0 to 133 points. Higher scores indicate better cognitive function.

#### Clinician Interview–Based Impression of Severity and Clinician Interview–Based Impression of Change Plus Caregiver Input

The Clinician Interview–Based Impression tool is used to assess the severity of cognitive function through semistructured interviews with patients and their caregivers [31]. The Clinician Interview–Based Impression of Severity (CIBIS) is specifically designed to assess baseline severity. Clinicians evaluate responses across 4 domains—general condition, mental or cognitive state, behavior, and ADL—on a scale of 1 to 7 points, where higher scores indicate greater severity. Clinician Interview–Based Impression of Change Plus Caregiver Input (CIBIC-Plus) is used during follow-up to evaluate whether there has been an improvement or decline compared to the baseline level. It assesses 3 aspects: cognition, behavior, and function. Based on these evaluations, the clinician rates the patient’s overall condition on a 7-point Likert scale, where 1 indicates very much improved and 7 indicates marked worsening.

#### Alzheimer’s Disease Cooperative Study-Mild Cognitive Impairment-Activities of Daily Living

The Alzheimer’s Disease Cooperative Study-Mild Cognitive Impairment-Activities of Daily Living (ADCS-MCI-ADL) assesses the ability of patients to perform ADL through a structured questionnaire administered to the patient and caregiver by a qualified rater [32]. The scale evaluates both basic and instrumental ADL, with the 24-item version used in this study. Scores range from 0 to 69, with higher scores indicating better ability to independently manage daily living activities, while lower scores reflect a higher degree of dependency when performing such tasks.

#### EQ-5D-3L and EQ-5D-Visual Analogue Scale

EQ-5D is a tool used to measure health-related quality of life, assessing the 5 dimensions of mobility, self-care, usual activities, pain or discomfort, and anxiety or depression [33]. EQ-5D-3L evaluates each of these dimensions on 3 levels: no problems, some problems, and extreme problems. The responses are then converted into an index score by applying weighted values to each item and summing them, resulting in a score ranging from 0 (death) to 1 (perfect health). In addition, EQ-5D-Visual Analogue Scale (EQ-5D-VAS) captures the respondent’s overall assessment of their health on a scale ranging from 0 (worst health imaginable) to 100 (best health imaginable). In this study, both EQ-5D-3L and EQ-5D-VAS were administered to assess the quality of life of patients with MCI as well as the quality of life of their study partners who live with them.

#### Safety Measures

The occurrence of AEs was monitored actively and systematically, following the Consolidated Standards of Reporting Trials (CONSORT) Harms guideline [34]. AEs were assessed weekly by investigators through telephone contact. Additionally, participants were allowed to report AEs spontaneously at any time during the trial. AEs that involve death, life-threatening conditions, hospitalization or prolonged hospitalization, persistent or significant disability or functional impairment, congenital anomalies or birth defects, or other medically important conditions were classified as serious adverse events (SAEs). Furthermore, the relationship between these AEs and the digital therapeutic device used in this trial was evaluated using a 5-level categorical scale ranging from “Definitely not related” to “Definitely related.”

### Outcomes

For the primary outcome, participants were categorized as responders or nonresponders based on whether their ADAS-Cog-14 scores were maintained or improved, and the proportion of responders was compared between the ET-101 and sham control groups at weeks 12 and 24. For the secondary outcomes, changes from baseline to 12 and 24 weeks were compared between groups using the following assessments: cognitive function (ADAS-Cog-14, K-MMSE-II, CDR-SB, ADCOMS, and DSC), ADL (CIBIS, CIBIC-Plus, and ADCS-MCI-ADL), and quality of life (EQ-5D-3L and EQ-5D-VAS). For the exploratory analysis, group comparisons of the change scores of the ADAS-Cog-14 subdomains were conducted, including the memory, language, and praxis subdomains. For the safety analysis, the proportions of total AEs, SAEs, and events deemed related to the digital therapeutic intervention were compared between groups.

### Statistical Analysis

All statistical analyses were conducted on the full analysis set population, which included all randomized participants who received at least 1 session of the assigned study treatment. This approach is consistent with the modified intention-to-treat concept used in many previous clinical trials [35-37]. Baseline characteristics were compared between the intervention and control groups using an independent 2-tailed *t* test for continuous variables and either a chi-square test or Fisher exact test for categorical variables. The primary outcome, the proportion of responders and nonresponders between the ET-101 group and the control group, was compared by means of a chi-square test. In this study, nonresponder imputation (NRI) was applied, classifying trial dropouts as nonresponders. NRI is a method of handling missing data by assigning all missing cases as nonresponders, thereby preventing treatment benefits from being overestimated. This approach is widely recognized and has been extensively adopted in clinical trials that investigate drug efficacy across various areas [38,39]. In this study, based on NRI, responders were defined as participants who completed the study through week 24 and whose ADAS-Cog-14 score was either maintained or decreased compared to the baseline level, indicating preserved or improved cognitive function [40]. In contrast, nonresponders included those who either failed to complete the study through week 24 or whose ADAS-Cog-14 score increased compared to the baseline level.

Secondary outcomes, in this case, clinical measures of cognition, ADL, and quality of life, were assessed by analyzing the changes from baseline to week 12 and week 24. Missing data in clinical scales due to trial dropouts were replaced using conditional mean imputation via a regression analysis. For the regression analysis used in imputation, the ADAS-Cog-14, K-MMSE-II, and CDR-SB scores at baseline, as well as the visit and visit-specific values of each clinical measure, were considered. Conditional mean imputation through a regression analysis was conducted using Python (version 3.9.20; Python Software Foundation). Independent *t* tests were used to evaluate whether the score changes differed significantly between the 2 groups. Additionally, a linear mixed model analysis was conducted to examine the treatment-time interaction for each clinical assessment. In this model, treatment, time (indicating baseline, week 12, and week 24), treatment-time interaction, and the baseline score of each scale were included as fixed effects. If a significant difference in the treatment-time interaction between the ET-101 and control groups was found in the linear mixed model, a post hoc analysis was performed to identify the specific time points at which the treatment-time interaction was significant. As a sensitivity analysis, to confirm that the imputation of missing data did not affect the results, the same linear mixed model analysis and post hoc analysis were performed without missing value imputation for variables where a significant treatment-time interaction was found in secondary outcomes.

As an exploratory analysis, the linear mixed model was applied to assess which subdomains of ADAS-Cog-14 (memory, language, and praxis) showed significant treatment-time interaction. If a significant treatment-time interaction was present, a post hoc analysis was conducted to determine the specific time points at which this interaction was significant. As a safety analysis, independent *t* tests were performed for continuous variables, while chi-square tests or Fisher exact tests were used for categorical variables.

All statistical tests were 2-tailed, and *P* scores of <.05 were considered statistically significant. For the post hoc analysis of the linear mixed model, Bonferroni-adjusted *P* scores were reported, with values <.05 indicating statistical significance. All statistical analyses were performed using the *lmerTest* package for the linear mixed model in R software (version 4.4.2; The R Foundation for Statistical Computing).

### Ethical Considerations

All participants provided written, informed consent to participate, and their study partners were asked to provide separate written, informed consent. The study was conducted following the Declaration of Helsinki and was approved by the institutional review boards (IRBs) at each of the 7 participating medical centers (Gachon University Gil Medical Center IRB: GCIRB2023-048; Konkuk University Medical Center IRB: 2023-02-017; The Catholic University of Korea, Yeouido St. Mary’s Hospital IRB: SC23DDDS0007; Severance Hospital IRB: 10-2023-68; The Catholic University of Korea, Eunpyeong St. Mary’s Hospital IRB: PC23DDDS0021; Kangwon National University Hospital IRB: 2023-02-001; SMG-SNU Boramae Medical Center IRB: 1-2023-0005). All participants were anonymized by assigning randomly generated identification numbers. All participants received a compensation of KRW 100,000 (US $68.22) per visit, and study partners were provided with KRW 50,000 (US $34.11) per visit. The study consisted of 5 visits for those who completed the entire study. The reporting of this trial follows the CONSORT statement (Multimedia Appendix 3).

## Results

### Overview

**Figure 1:**
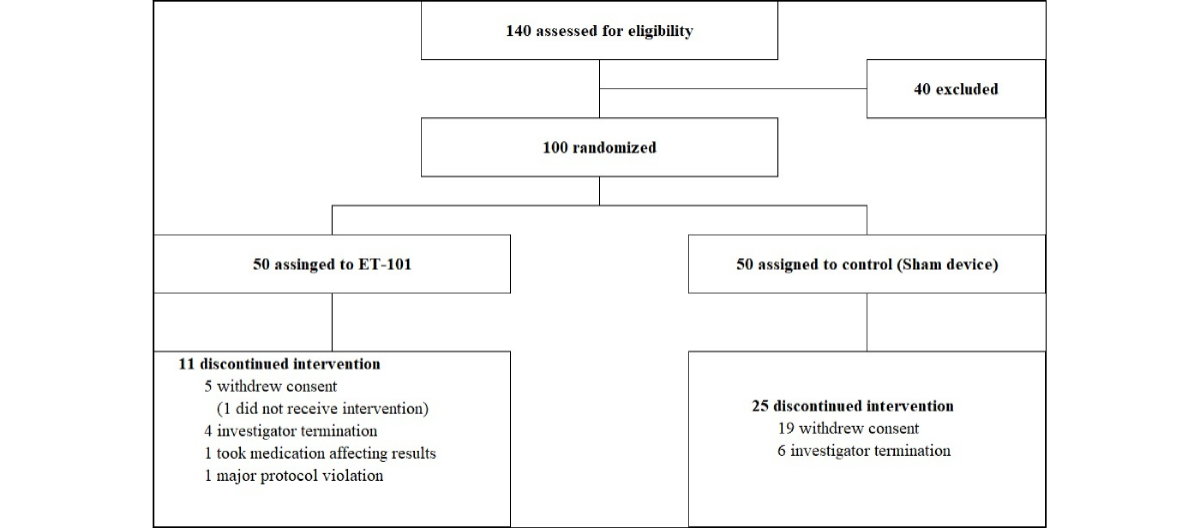
Flowchart of the study process.

A total of 140 participants were screened, of whom 40 were excluded based on the inclusion and exclusion criteria (Figure 1). The remaining 100 participants were randomized at a 1:1 ratio. During the clinical trial period, 11 (22.4%) participants in the ET-101 group and 25 (50%) participants in the control group did not complete the study. In the ET-101 group, 1 participant did not use the digital therapeutic device at all after randomization. Therefore, the analysis was conducted on a total of 99 participants in the full analysis set. The dropout rate from the beginning of the training to week 12 was 3 (6%) participants in the ET-101 group and 8 (16%) participants in the sham control group. From week 12 to week 24, the dropout rate was 8 (16%) participants in the ET-101 group and 17 (34%) participants in the sham control group. The adherence rate, defined as the proportion of completed sessions during the 12-week intervention period, was higher in the ET-101 group (mean 83.2%, SD 24.4%) compared to the sham control group (mean 63.8%, SD 31.4%). Daily usage, measured by the number of days participants accessed the program out of the total 84 scheduled training days (12 weeks × 7 days), was also higher in the ET-101 group (mean 69.7, SD 20.2 days) than in the sham control group (mean 53.3, SD 25.8 days). The baseline demographic and clinical characteristics are summarized in Table 1. There were no statistically significant differences in baseline characteristics between the 2 groups.

**Table 1:** Baseline characteristics of study participants.

Characteristics		ET-101 (n=49)	Control (n=50)	*P* value	
Age (years), mean (SD)		75 (5.77)	74.8 (5.79)	.84	
**Sex, n (%)**					.91
	Male	21 (42.9)	22 (44)		
	Female	28 (57.1)	28 (56)		
Education (years), mean (SD)		10.9 (4.47)	10.1 (4.82)	.34	
**Current medical illness, n (%)**					.96
	Yes	37 (75.5)	38 (76)		
	No	12 (24.5)	12 (24)		
**Current use of** **acetylcholinesterase inhibitors or memantine** **, n (%)**					.90
	Yes	18 (36.7)	19 (38)		
	No	31 (63.3)	31 (62)		
ADAS-Cog-14^a^, mean (SD)^b^		29.2 (6.88)	31 (6.23)	.17	
K-MMSE-II^c^, mean (SD)		23.9 (2.79)	23 (2.7)	.12	
CDR-SB^d^, mean (SD)^b^		1.66 (0.856)	2.03 (0.997)	.05	
ADCOMS^e^, mean (SD)^b^		0.295 (0.146)	0.347 (0.128)	.07	
DSC^f^, mean (SD)		38.2 (15.4)	34.7 (17.1)	.29	
CIBIS^g^, mean (SD)^a^		2.67 (0.516)	2.82 (0.523)	.16	
ADCS-MCI-ADL^h^, mean (SD)		44.3 (6.59)	41.6 (7.06)	.05	
EQ-5D-3L patient, mean (SD)		0.929 (0.090)	0.909 (0.087)	.27	
EQ-5D-3L study partner, mean (SD)		0.926 (0.092)	0.946 (0.079)	.25	
EQ-5D-VAS^i^ patient, mean (SD)		78.6 (13)	77.3 (18.7)	.70	
EQ-5D-VAS study partner, mean (SD)		76.8 (13.6)	82.2 (14)	.06	

^a^ADAS-Cog-14: Alzheimer’s Disease Assessment Scale-Cognitive Subscale-14.

^b^Lower scores represent better performance.

^c^K-MMSE-II: Korean Mini Mental State Examination, 2nd Edition.

^d^CDR-SB: Clinical Dementia Rating-Sum of Boxes.

^e^ADCOMS: Alzheimer’s Disease Composite Score.

^f^DSC: Digit Symbol Coding.

^g^CIBIS: Clinician Interview–Based Impression of Severity.

^h^ADCS-MCI-ADL: Alzheimer’s Disease Cooperative Study-Mild Cognitive Impairment-Activities of Daily Living.

^i^EQ-5D-VAS: EQ-5D-Visual Analogue Scale.

### Primary Outcome

As shown in Table 2, the proportion of responders and nonresponders did not differ significantly between the ET-101 group and the control group at week 12 (*P*=.47). However, at visit 4, the proportion of responders was significantly higher in the ET-101 group (n=29, 59.2% responders) compared to the sham device group (n=14, 28% responders; *P*=.002).

**Table 2:** Comparison of the proportion of responders and nonresponders between the ET-101 and control groups.

			ET-101 (n=49), n (%)		Control (n=50), n (%)		*P* value	
**12 weeks**								.47
	Responder	20 (40.8)		24 (48)				
	Nonresponder	29 (59.2)		26 (52)				
**24 weeks**								.002
	Responder	29 (59.2)		14 (28)				
	Nonresponder	20 (40.8)		36 (72)				

### Secondary Outcome

**Figure 2:**
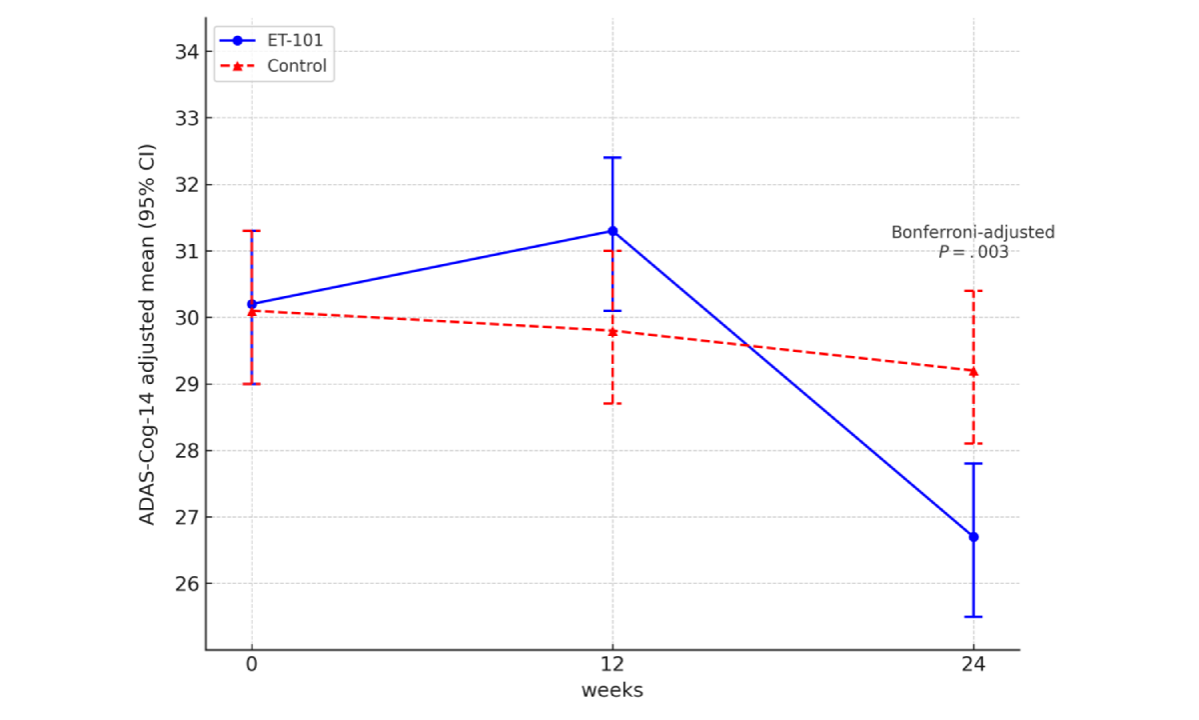
Comparison of adjusted mean change of Alzheimer’s Disease Assessment Scale-Cognitive Subscale-14 (ADAS-Cog-14) between ET-101 and the control group using a linear mixed model. Lower scores represent better performance. ADAS-Cog-14 scores adjusted for baseline differences.

The changes in clinical measures from baseline to weeks 12 and 24 are presented in Table 3. For ADAS-Cog-14, the change in scores from baseline to week 12 was compared between the ET-101 and control groups, showing no statistically significant differences (change difference=1.42; *P*=.13). However, at week 24, the change in scores from baseline showed a statistically significant improvement in the ET-101 group compared to the control group (change difference=–2.55; *P*=.02). These findings remained consistent even when examining the treatment-time interaction effect in a linear mixed model that adjusted for baseline ADAS-Cog-14 scores (*F*_2, 194_=7.45; *P*<.001). In the post hoc analysis conducted to identify the specific time points associated with the significant treatment-time interaction, no significant differences were observed between the 2 groups at baseline (estimates=0.022; t_265_=0.026; Bonferroni-adjusted *P*=.98) or week 12 (estimates=1.44; t_265_=1.74; Bonferroni-adjusted *P*=.08). However, at week 24, cognitive improvement as measured by ADAS-Cog-14 was statistically significant in the ET-101 group compared to the control group (estimates=–2.53; t_265_=–3.05; Bonferroni-adjusted *P*=.003; Figure 2). For the other clinical measures, no statistically significant differences were observed.

**Table 3:** Comparison of cognition, activities of daily living, and quality of life questionnaires between the ET-101 and control groups.

Questionnaire and week		ET-101 (n=49), mean (SD)	Control (n=50), mean (SD)	Change difference^a^ (95% CI)	Linear mixed model (treatment-time interaction)			
					*F* test (*df*)		*P* value	
**ADAS-Cog-14^b,c^**						7.45 (2, 194)		<.001
	Week 12	30.3 (7.81)	30.7 (7.40)	1.42 (–0.44 to 3.29)				
	Week 24	25.8 (9.52)	30.1 (8.55)	–2.55 (–4.69 to –0.417)^d^				
**K-MMSE-II^e^**						0.454 (2, 194)		.64
	Week 12	24.1 (3.34)	23.6 (2.82)	–0.377 (–1.38 to 0.631)				
	Week 24	23.6 (3.16)	23.1 (2.74)	–0.386 (–1.47 to 0.703)				
**CDR-SB^b,f^**						0.865 (2, 194)		.42
	Week 12	1.68 (1.00)	1.92 (1.04)	0.130 (–0.111 to 0.372)				
	Week 24	1.43 (1.01)	1.85 (1.02)	–0.055 (–0.381 to 0.272)				
**ADCOMS^b,g^**						0.608 (2, 194)		.55
	Week 12	0.315 (0.175)	0.343 (0.154)	0.024 (–0.014 to 0.062)				
	Week 24	0.267 (0.180)	0.310 (0.161)	0.008 (–0.044 to 0.060)				
**DSC^h^**						0.781 (2, 194)		.46
	Week 12	39.1 (14.9)	34.8 (18.8)	0.798 (–1.34 to 2.94)				
	Week 24	39.0 (14.1)	33.9 (16.0)	1.62 (–1.29 to 4.52)				
**CIBIC-Plus^b,i^**						0.526 (1, 97)		.47
	Week 12	3.84 (0.717)	4.02 (0.553)	–0.183 (–0.439 to 0.073)^j^				
	Week 24	4.02 (0.433)	4.10 (0.364)	–0.080 (–0.239 to 0.080)^j^				
**ADCS-MCI-ADL^k^**						1.73 (2, 194)		.18
	Week 12	43.3 (5.78)	40.7 (6.41)	–0.101 (–1.73 to 1.53)				
	Week 24	43.2 (5.86)	41.9 (6.01)	–1.40 to (–3.16 to 0.352)				
**EQ-5D-3L patient**						0.963 (2, 194)		.38
	Week 12	0.925 (0.095)	0.908 (0.098)	–0.004 (–0.038 to 0.030)				
	Week 24	0.920 (0.108)	0.925 (0.085)	–0.024 (–0.060 to 0.012)				
**EQ-5D-3L study partner**						1.50 (2, 194)		.23
	Week 12	0.924 (0.101)	0.937 (0.106)	0.008 (–0.028 to 0.045)				
	Week 24	0.926 (0.096)	0.967 (0.056)	–0.021 (–0.050 to 0.009)				
**EQ-5D-VAS^l^ patient**						0.705 (2, 194)		.50
	Week 12	77.5 (15.9)	75.2 (15.8)	0.978 (–5.26 to 7.21)				
	Week 24	72.3 (15.4)	67.5 (13.8)	3.53 (–2.39 to 9.46)				
**EQ-5D-VAS study partner**						1.92 (2, 194)		.15
	Week 12	77.7 (11.6)	81.6 (13.0)	1.38 (–4.14 to 6.90)				
	Week 24	77.0 (10.7)	77.2 (12.0)	5.12 (–0.873 to 11.1)				

^a^The score change from baseline to weeks 12 and 24 in the ET-101 group minus the score change from baseline to weeks 12 and 24 in the control group.

^b^Lower scores represent better performance.

^c^ADAS-Cog-14: Alzheimer’s Disease Assessment Scale-Cognitive Subscale-14.

^d^*P*<.05.

^e^K-MMSE-II: Korean Mini Mental State Examination, 2nd Edition.

^f^CDR-SB: Clinical Dementia Rating-Sum of Boxes.

^g^ADCOMS: Alzheimer’s Disease Composite Score.

^h^DSC: Digit Symbol Coding.

^i^CIBIC-Plus: Clinician Interview–Based Impression of Change Plus Caregiver Input.

^j^Due to the inability to present mean change, the difference in mean change between the groups was replaced with score differences across time points.

^k^ADCS-MCI-ADL: Alzheimer’s Disease Cooperative Study-Mild Cognitive Impairment-Activities of Daily Living.

^l^EQ-5D-VAS: EQ-5D-Visual Analogue Scale.

### Sensitivity Analysis

Even when the linear mixed model was used without applying conditional mean imputation for missing data, the treatment-time interaction remained statistically significant in ADAS-Cog-14 (*F*_2, 176_=3.91; *P*=.02). In the post hoc analysis, no statistically significant differences were observed at baseline (estimates=0.018; t_232_=0.021; Bonferroni-adjusted *P*=.98) or at week 12 (estimates=1.53; t_235_=1.69; Bonferroni-adjusted *P*=.09). At week 24, the Bonferroni-adjusted *P* value was .05, indicating a trend toward greater improvement in ADAS-Cog-14 scores in the ET-101 group compared to those of the control group (estimates=–2.13; t_244_=–1.96).

### Exploratory Analysis

The results of the linear mixed model and post hoc analysis for the subdomains of ADAS-Cog-14 are presented in Table S4 in Multimedia Appendix 1. Among the subdomains of ADAS-Cog-14, the treatment-time interaction in the memory domain was statistically significant (*F*_2, 194_=11.2; *P*<.001). In the post hoc analysis, ET-101 showed a statistically significant improvement in the memory domain compared to the control group at week 24 (estimates=–2.50; t_264_=–4.03; Bonferroni-adjusted *P*<.001).

The treatment-time interaction was also found to be statistically significant for the language domain (*F*_2, 194_=3.99; *P*=.02). In the post hoc analysis, ET-101 demonstrated a statistically significant improvement compared to the control group at week 24 (estimates=–0.807; t_290_=–3.68; Bonferroni-adjusted *P*<.001). For the praxis subdomain, representing executive function, the treatment-time interaction was not statistically significant (*F*_2, 194_=0.497; *P*=.67).

### Safety Analysis

In the ET-101 group, 6 AEs occurred, while 4 AEs were reported in the control group, with no statistically significant difference in AE incidence between the groups (*P*=.53; Table 4). Among the AEs in the ET-101 group, 2 were classified as SAEs due to prolonged hospitalization (non-ST elevation myocardial infarction and herniated disc disease of the lumbar spine); however, the incidence of SAEs also did not differ significantly between the 2 groups (*P*=.47). All reported AEs were determined to be definitely not related to ET-101.

**Table 4:** Comparison of safety outcomes between ET-101 and the control group.

		ET-101 (n=49), n (%)	Control (n=50), n (%)	*P* value	
**Serious adverse events**					.47
	Yes	2 (4.1)	0 (0)		
	No	4 (8.2)	4 (8)		
**Relevance to digital therapeutics**					N/A^a^
	Definitely related	0 (0)	0 (0)		
	Probably related	0 (0)	0 (0)		
	Possibly related	0 (0)	0 (0)		
	Probably not related	0 (0)	0 (0)		
	Definitely not related	6 (12.2)	4 (8)		
	Unknown	0 (0)	0 (0)		
Total		6 (12.2)	4(8)	.52	

^a^N/A: not applicable.

## Discussion

### Principal Findings

This study investigated the efficacy and safety of ET-101 in patients with MCI compared to a sham device control group. We found that the ET-101 group showed significantly greater improvement in the ADAS-Cog-14 score in 24 weeks compared to the control group. Furthermore, no AEs related to the use of ET-101 were observed. Among the various types of digital cognitive training, to the best of our knowledge, this study is the first to investigate the effects of MMT using multimemory strategies in the form of a mobile app.

ET-101, a metamemory-based multistrategic cognitive training program, showed improvement in global cognitive function as well as memory function. In the primary outcome, the responder rate at 24 weeks was significantly higher in the ET-101 group. Additionally, in the secondary outcomes, the ADAS-Cog-14 score showed a statistically significant improvement in the ET-101 group compared to the control group at 24 weeks. According to a meta-analysis that included trials on computerized cognitive training for individuals with MCI, independently delivered digital cognitive training programs, such as ET-101, demonstrated a significant effect on verbal episodic memory, with a standardized mean difference of 0.21 (95% CI 0.04-0.38). This is consistent with the observed memory improvement in this study [41]. ET-101 demonstrated cognitive improvement comparable to that observed with donepezil in terms of ADAS-Cog score changes, suggesting that ET-101 may have a similar cognitive improvement effect [42]. One of the most well-known protective factors against memory decline in MCI is cognitive reserve, which refers to the ability to use cognitive processes and brain networks adaptively to compensate for deterioration [43]. In MCI, there is not only a reduction in the hippocampal volume but also a weakening of the connectivity among key brain regions involved in memory function, such as the frontal, temporal, and parietal lobes [44]. With ET-101, MMT enables individuals to monitor their own memory function objectively and select the most efficient memory strategies tailored to their needs, facilitating the frontal lobe [14]. Additionally, efficient memory strategies, including attention, imagination, and association, enhance the connectivity of brain regions related to cognitive reserve [23,24]. Biological evidence also supports the cognitive reserve–enhancing effects of metamemory. A previous study of MMT found reduced mean diffusivity in the left superior longitudinal fasciculus and corona radiata, which link the frontal, temporal, and parietal lobes [14], suggesting that ET-101 may contribute to enhancing cognitive reserve, which in turn could support improvements in memory function.

Another characteristic of ET-101’s cognitive function improvement is that it has a transfer effect on the language domain. In the subdomain analysis of ADAS-Cog-14, the ET-101 group showed a significantly greater improvement in the language domain compared to the control group at 24 weeks. Meta-analyses of several studies of cognitive training in patients with MCI have not shown significant improvements in the language domain [41]. Earlier work has suggested that cognitive training gains typically reflect the training content and that insufficient training on other cognitive domains may therefore make improvements beyond the memory domain less observable [41]. However, MMT-based multistrategic cognitive training, on which ET-101 is based, has consistently demonstrated functional improvements not only in memory but also in the language domain across multiple studies [11,12,14]. One possible explanation for this transfer effect is that the metamemory and memory strategies of ET-101 repeatedly strengthen the connection between episodic memory and semantic memory. MMT helps individuals select compensatory strategies to address their cognitive deficits, and ET-101, in particular, repeatedly trains strategies such as imagination and association, facilitating the connection between episodic and semantic memory [45]. This is especially relevant to lexical production, which is impaired in MCI and requires effortful semantic memory processing, such as in fluency and naming tasks [46]. Notably, naming and semantic fluency have been identified in the literature as strong predictors of the conversion from MCI to Alzheimer dementia, and ET-101 appears to reinforce cognitive functions that are closely associated with this deterioration [21]. Additional long-term studies are needed to investigate the effects of ET-101 on the rate of conversion from MCI to Alzheimer dementia and to assess its long-term efficacy further.

The observed improvement in the memory and language domains following ET-101 training is closely linked to the memory strategies embedded in the program, which are known to support memory formation. ET-101 primarily incorporates 3 core memory strategies: attention, imagination, and association. First, attention serves as an initial step in memory formation by facilitating selective encoding. Numerous neuroimaging studies have demonstrated that attention enhances memory by activating interactions between the hippocampus and fronto-parietal regions within the dorsal attention network [47-49]. This activation is thought to strengthen neocortical representations, thereby supporting long-term memory storage [50]. Clinically, studies have shown that increased activation of the dorsal attention network is positively correlated with the accuracy and strength of memory recall [51]. Next, imagination shares common neural circuits with episodic memory, involving the hippocampus, medial prefrontal cortex, and anterolateral temporal cortex—regions that are closely associated with memory consolidation [52,53]. Hippocampal replay facilitates the reconstruction of sensory experiences by integrating visual, sensory, and semantic information [52]. This process allows imagination to contribute to memory formation and strengthening, even in the absence of direct sensory input [52]. Regarding association, it has been suggested that newly learned words can facilitate the recall of previously acquired information through retroactive facilitation [54]. Notably, neural evidence indicates that while preexisting semantic relationships are primarily represented within the neocortex, unrelated word pairs can be bound by the hippocampus through associative processes, along with their episodic list context and novelty [54]. Taken together, findings from previous studies suggest that the strategies used in ET-101—attention, imagination, and association—effectively activate the hippocampus and related regions, thereby potentially compensating for the memory deficits observed in individuals with MCI.

However, ET-101 did not show a significant difference compared to the control group on the praxis subdomain, ADL, or quality of life measures. There was no statistically significant difference between the ET-101 and control groups in ADL assessments using CIBIC-Plus and ADCS-MCI-ADL, or in quality-of-life assessments for patients with MCI and their study partners using EQ-5D. Additionally, the praxis domain of ADAS-Cog-14 did not show a significant difference between the groups up to 24 weeks. These findings appear to be related to the characteristics of MCI and the measured scales. MCI involves cognitive decline, but the functional impairment at this stage is not severe enough to warrant a diagnosis of overt dementia [55,56]. Given that those with MCI typically manage daily activities well, ADL and quality-of-life scales may have a ceiling effect, reducing their sensitivity to detect meaningful changes [57]. Therefore, to observe subtle changes in ADL among individuals with MCI, this study used the ADCS-MCI-ADL scale, which is sensitive to detecting changes in ADL among individuals with MCI [32]. In prior pharmacological trials, reported improvements in ADCS-MCI-ADL included participants with both MCI and mild Alzheimer disease [3,58]. However, such improvements in ADCS-MCI-ADL have typically been observed over longer durations, such as 18-month follow-up periods, whereas the 24-week follow-up in our study may not have been long enough to detect meaningful changes [3,58]. This suggests that longer-term follow-up may be necessary to capture significant improvements in ADL or quality of life associated with ET-101. Similarly, in ADAS-Cog-14, the praxis domain has been shown to be less effective in distinguishing different stages of cognitive decline compared to the memory and language domains, and it appears to exhibit a ceiling effect in MCI [26]. Given recent efforts to develop more sensitive scales for detecting changes in ADL, quality of life, and cognitive function in MCI, future studies using these updated tools will be essential for accurately evaluating the effects of ET-101.

The improvement in cognitive function with ET-101 followed a delayed pattern, with significant effects emerging at the 24-week follow-up. While no group differences were observed immediately after the 12-week training period in either the primary or secondary outcomes, the ET-101 group showed significantly greater responder rates and ADAS-Cog-14 improvements at 24 weeks. These findings suggest that the cognitive benefits of ET-101 may not be immediate but instead become evident and persist after the intervention ends. This pattern contrasts with traditional cognitive training, which often shows immediate but short-lived effects. For example, the Advanced Cognitive Training for Independent and Vital Elderly trial reported improvements immediately after memory training, but these effects were not maintained at the 1-year follow-up [7]. In a study of computerized cognitive training that included follow-up data, the initially significant improvements in attention and memory observed immediately after the intervention were no longer present at the 3-month follow-up [8]. Previous meta-analyses have suggested that the effects of memory strategy training tend to be short-lived [59]. Moreover, most studies have not evaluated maintenance effects, resulting in limited evidence on whether training-induced improvements observed during the intervention are sustained in real-life situations [15]. In contrast, ET-101 showed significant gains after the training ended. This delayed onset and sustained improvement align with findings from strategy-based training programs, such as those including memory education, skill practice, and cognitive restructuring of memory-related beliefs [60-62]. Similar to ET-101, these studies did not show immediate cognitive improvements posttraining but demonstrated significant effects during follow-up assessments [60-62]. Such outcomes suggest that benefits emerge gradually as participants apply learned strategies in real-world contexts [61-63]. From a neurobiological perspective, memory consolidation involves synaptic consolidation, which includes short-term molecular processes such as protein synthesis and synaptic potentiation occurring within minutes to hours after memory formation, followed by systems consolidation, during which the interaction between the hippocampus and neocortex is gradually strengthened [64,65]. System consolidation can take several months to years in humans, and sufficient time may be required to detect changes in memory ensembles and their associated engram networks, which could explain the delayed onset of cognitive improvement observed in this study [65]. However, alternative explanations cannot be excluded. The cognitive improvement observed at the 24-week follow-up may also be partially influenced by practice effects in cognitive testing or by reduced psychological distress after the completion of trial participation, leading to more apparent posttrial improvements. Moreover, the underlying mechanisms of the delayed and lasting cognitive effects remain unclear, and further longitudinal follow-up and functional neuroimaging studies are warranted to elucidate the temporal dynamics of training-induced neural plasticity. Future meta-analyses and systematic reviews should also investigate the characteristics of cognitive training protocols associated with either immediate or delayed effects to clarify the factors that determine these distinct temporal response patterns.

The cognitive improvement effect of ET-101 was observed in the ADAS-Cog-14 case, but not on other cognitive function assessment scales. In the secondary outcomes, the measures K-MMSE-II, CDR-SB, ADCOMS, and DSC did not show a statistically significant treatment-time interaction. These findings may be related to the sensitivity of cognitive function assessment scales. Previous studies comparing ADAS-Cog-14 with other cognitive measures have shown that ADAS-Cog can detect cognitive changes in MCI significantly more sensitively compared to MMSE and CDR-SB [66,67]. Notably, this study used the 14-item version of ADAS-Cog, which has been specifically reported to be highly sensitive for MCI assessment [26]. Therefore, the observed improvement in ADAS-Cog-14, but not on other scales, is likely due to differences in the sensitivity of these assessments in MCI, with ADAS-Cog-14 being more capable of detecting subtle cognitive changes.

### Limitations

One limitation of this study is the relatively high number of dropouts during the trial. During the 12-week training period, 3 (6%) participants in the ET-101 group and 8 (16%) participants in the sham control group dropped out, values that fall within or below the typical range of dropout rates (30%-40%) reported in previous digital intervention studies [68,69]. However, between week 12 and week 24—after the intervention had ended—additional dropouts occurred in both groups: 8 (16%) participants in the ET-101 group and 17 (34%) in the sham control group. This increase may reflect reduced motivation to continue participation in the absence of active training. As these attrition rates necessitated an imputation strategy for missing data, the interpretation of the results should be approached with caution. In particular, the NRI method may overpenalize attrition and potentially bias the treatment effect estimates downward, especially when the reasons for dropout differ between groups. Therefore, to minimize the impact of the imputation used in this study, we conducted analyses to verify whether the results remained consistent across different statistical approaches. These included a comparison of the proportion of participants showing maintenance or improvement in cognitive function between the ET-101 and sham control groups based on NRI, as well as analyses of continuous cognitive scores using both independent *t* tests and linear mixed models. Furthermore, to minimize the impact of the imputation as conducted here, we used the same linear mixed model without imputation for missing data. Across these multiple analytic approaches, we consistently observed that ET-101 produced significantly better maintenance or improvement of cognitive function compared to sham controls at the 24-week follow-up.

Additionally, certain clinical variables, in this case CDR-SB and EQ-5D-VAS, had *P* values close to .05 in baseline comparisons, indicating that the 2 groups were not entirely homogeneous in terms of certain variables. To address this, we incorporated each scale’s baseline score as a fixed effect in the linear mixed model when analyzing secondary outcomes. This approach allowed us to confirm that the treatment-time interaction remained statistically significant even after adjusting for these differences.

Third, although participants were not explicitly informed of their group assignment, differences in content between the ET-101 and sham control groups may have caused some participants to infer their allocation based on the app experience. This is a common issue in studies evaluating the efficacy of software-based interventions, where a sham condition must be provided without including the active training components. As such, there is a potential risk of performance and expectation bias between groups, which must be taken into consideration when interpreting the results.

Fourth, this study included only participants who were able to own and operate a smartphone, which may have led to the inclusion of a relatively more tech-savvy population. However, this inclusion criterion was necessary because ET-101 is designed to be used independently without additional assistance during the training process, targeting individuals with basic smartphone literacy. In particular, in the context of Korea, epidemiological data from 2023 show that approximately 95% of individuals aged 60 years and older own a personal mobile phone [70]. Therefore, this inclusion criterion does not substantially deviate from the characteristics of the older adult population in Korea.

### Conclusions

Metamemory-based multistrategic cognitive training, ET-101, was found to improve cognitive function compared to a sham device control group. This effect was observed in both the targeted domain of memory and in the language domain, indicating a transfer effect. Additionally, the cognitive improvement became pronounced at 24 weeks, suggesting that ET-101 has a sustained effect beyond the training period. These findings indicate that ET-101 has the potential to provide effective MMT to a broader population with MCI, overcoming location and personnel limitations via its mobile app–based platform.

## Appendix

Multimedia Appendix 1 Supplementary tables.

Multimedia Appendix 2 Sample exercise from the ET-101 cognitive training program.

Multimedia Appendix 3 CONSORT-EHEALTH checklist (V 1.6.1).

## Abbreviations

ADAS-Cog: Alzheimer’s Disease Assessment Scale-Cognitive Subscale
ADCOMS: Alzheimer’s Disease Composite Score
ADCS-MCI-ADL: Alzheimer’s Disease Cooperative Study-Mild Cognitive Impairment-Activities of Daily Living
ADL: activities of daily living
AE: adverse event
CDR-SB: Clinical Dementia Rating-Sum of Boxes
CERAD-NP: Consortium to Establish a Registry for Alzheimer’s Disease Neuropsychological Assessment Battery
CIBIC-Plus: Clinician Interview–Based Impression of Change Plus Caregiver Input
CIBIS: Clinician Interview–Based Impression of Severity
CONSORT: Consolidated Standards of Reporting Trials
DSC: Digit Symbol Coding
EQ-5D-VAS: EQ-5D-Visual Analogue Scale
IRB: institutional review board
K-MMSE-II: Korean Mini Mental State Examination, 2nd Edition
MCI: mild cognitive impairment
MMSE: Mini Mental State Examination
MMT: metamemory training
NRI: nonresponder imputation
SAE: serious adverse event
SNSB: Seoul Neuropsychological Screening Battery

## Data Availability

The datasets analyzed during this study are available from the corresponding author on reasonable request.
