# Supplementary material for: Efficacy and Safety of Mobile App–Based Metamemory Cognitive Training for Mild Cognitive Impairment: Multicenter Randomized Clinical Trial
Source: JMIR Mhealth Uhealth. 2026 Jan 19;14:e73464. doi: 10.2196/73464 (PMC12865355; doi:10.2196/73464)
Supplement: Multimedia Appendix 2 [file mhealth_v14i1e73464_app2.pdf]

Sample Exercise from the ET-101 Cognitive Training Program

1. Training Screen Example – Korean Version

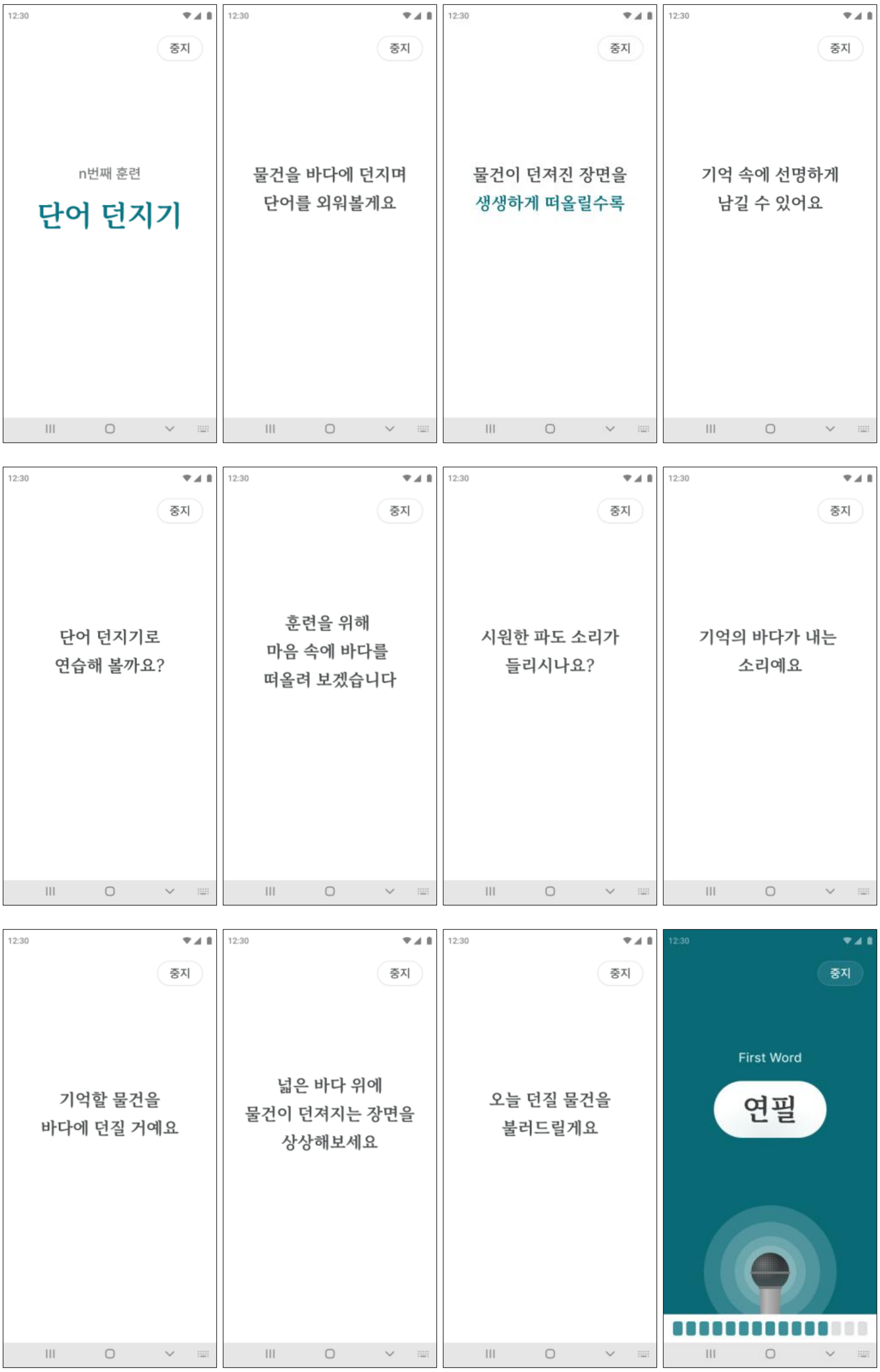

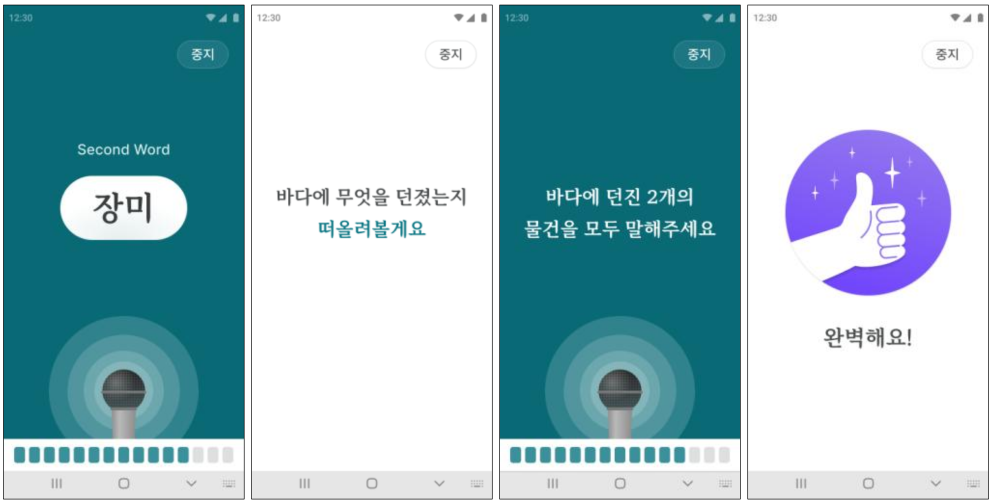

2. Training Screen Example – English Version (The actual clinical trial was conducted in Korean. The English version is provided for reference.)

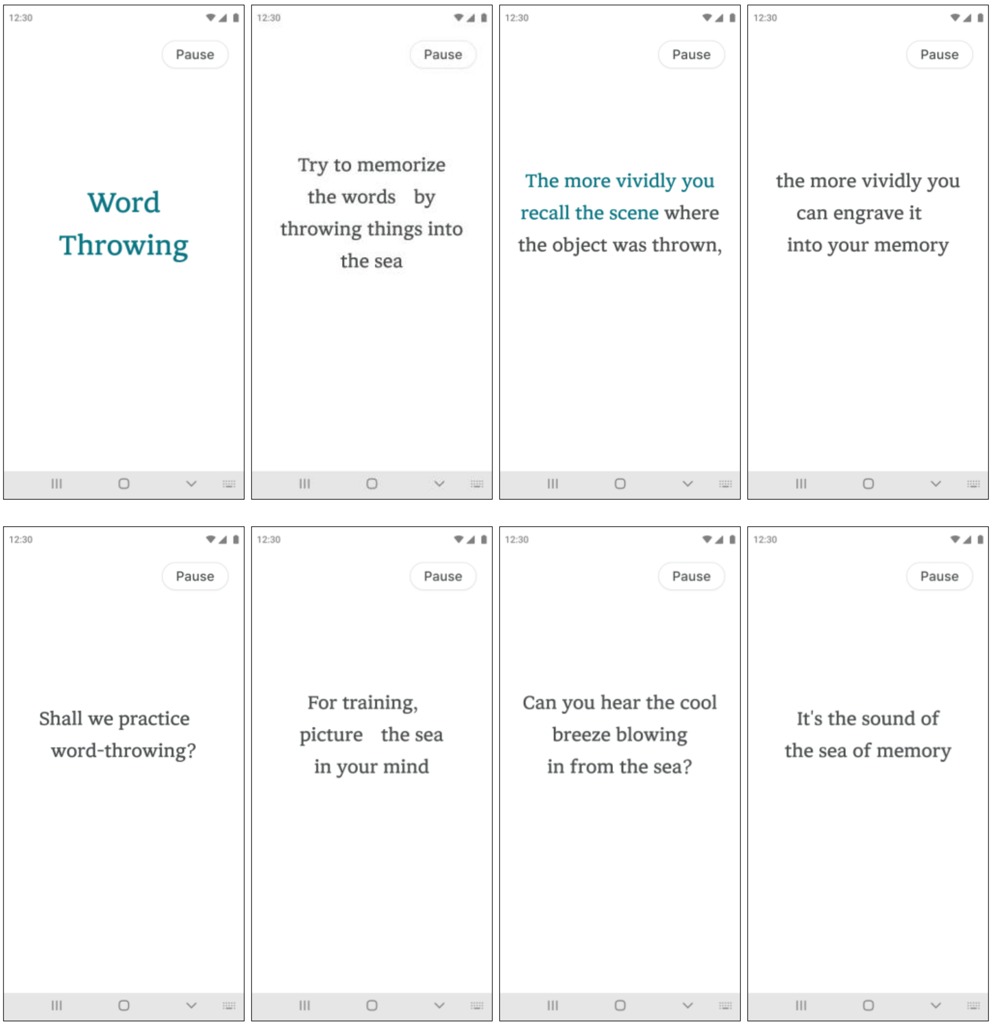

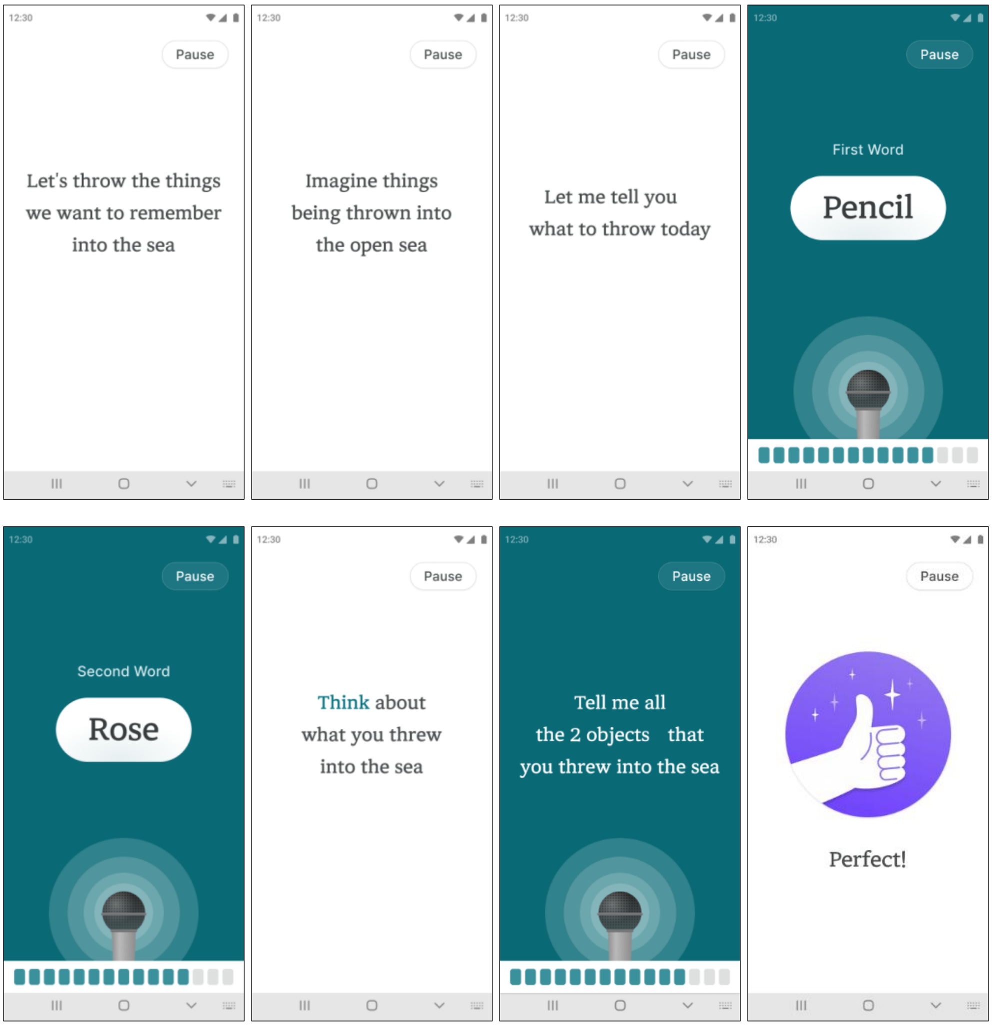
12:30
Pause
Let's throw the things we want to remember into the sea
Imagine things being thrown into the open sea
Let me tell you what to throw today
First Word
Pencil
Second Word
Rose
Think about what you threw into the sea
Tell me all the 2 objects that you threw into the sea
Perfect!
