# Supplementary material for: Efficacy and Safety of Mobile App–Based Metamemory Cognitive Training for Mild Cognitive Impairment: Multicenter Randomized Clinical Trial
Source: JMIR Mhealth Uhealth. 2026 Jan 19;14:e73464. doi: 10.2196/73464 (PMC12865355; doi:10.2196/73464)
Supplement: Multimedia Appendix 3 [file mhealth_v14i1e73464_app3.pdf]

CONSORT-EHEALTH (V 1.6.1) - Submission/Publication Form

The CONSORT-EHEALTH checklist is intended for authors of randomized trials evaluating web-based and Internet-based applications/interventions, including mobile interventions, electronic games (incl multiplayer games), social media, certain telehealth applications, and other interactive and/or networked electronic applications. Some of the items (e.g. all subitems under item 5 - description of the intervention) may also be applicable for other study designs.

The goal of the CONSORT EHEALTH checklist and guideline is to be
a) a guide for reporting for authors of RCTs,
b) to form a basis for appraisal of an ehealth trial (in terms of validity)

CONSORT-EHEALTH items/subitems are MANDATORY reporting items for studies published in the Journal of Medical Internet Research and other journals / scientific societies endorsing the checklist.

Items numbered 1., 2., 3., 4a., 4b etc are original CONSORT or CONSORT-NPT (non-pharmacologic treatment) items.
Items with Roman numerals (i., ii, iii, iv etc.) are CONSORT-EHEALTH extensions/clarifications.

As the CONSORT-EHEALTH checklist is still considered in a formative stage, we would ask that you also RATE ON A SCALE OF 1-5 how important/useful you feel each item is FOR THE PURPOSE OF THE CHECKLIST and reporting guideline (optional).

Mandatory reporting items are marked with a red *.
In the textboxes, either copy & paste the relevant sections from your manuscript into this form - please include any quotes from your manuscript in QUOTATION MARKS,
or answer directly by providing additional information not in the manuscript, or elaborating on why the item was not relevant for this study.

YOUR ANSWERS WILL BE PUBLISHED AS A SUPPLEMENTARY FILE TO YOUR PUBLICATION IN JMIR AND ARE CONSIDERED PART OF YOUR PUBLICATION (IF ACCEPTED).
Please fill in these questions diligently. Information will not be copyedited, so please use proper spelling and grammar, use correct capitalization, and avoid abbreviations.

DO NOT FORGET TO SAVE AS PDF _AND_ CLICK THE SUBMIT BUTTON SO YOUR ANSWERS ARE IN OUR DATABASE !!!

Citation Suggestion (if you append the pdf as Appendix we suggest to cite this paper in the caption):
Eysenbach G, CONSORT-EHEALTH Group
CONSORT-EHEALTH: Improving and Standardizing Evaluation Reports of Web-based and Mobile Health Interventions
J Med Internet Res 2011;13(4):e126

URL: http://www.jmir.org/2011/4/e126/
doi: 10.2196/jmir.1923
PMID: 22209829

sunny@emocog.com 계정 전환

비공개

초안 저장됨

* 표시는 필수 질문임

Your name *

First Last

Sunyoung Kang

Primary Affiliation (short), City, Country *

University of Toronto, Toronto, Canada

Department of Psychiatry, Yonsei University Cc

Your e-mail address *

abc@gmail.com

tjs4718@yuhs.ac

Title of your manuscript *

Provide the (draft) title of your manuscript.

The Efficacy and Safety of a Mobile App-Based Metamemory Cognitive Training for Mild Cognitive Impairment: Multicenter, Randomized Clinical Trial

Name of your App/Software/Intervention *

If there is a short and a long/alternate name, write the short name first and add the long name in brackets.

ET-101(Cogthera)

Evaluated Version (if any)

e.g. "V1", "Release 2017-03-01", "Version 2.0.27913"

Release 2023.06.08

Language(s) *

What language is the intervention/app in? If multiple languages are available, separate by comma (e.g. "English, French")

Korean

URL of your Intervention Website or App

e.g. a direct link to the mobile app on app in appstore (itunes, Google Play), or URL of the website. If the intervention is a DVD or hardware, you can also link to an Amazon page.

https://www.emocog.com/

URL of an image/screenshot (optional)

내 답변

Accessibility *

Can an enduser access the intervention presently?

- ○ access is free and open
- ◉ access only for special usergroups, not open
- ○ access is open to everyone, but requires payment/subscription/in-app purchases
- ○ app/intervention no longer accessible
- ○ 기타:

Primary Medical Indication/Disease/Condition *

e.g. "Stress", "Diabetes", or define the target group in brackets after the condition, e.g. "Autism (Parents of children with)", "Alzheimers (Informal Caregivers of)"

Mild Cognitive Impairment (Patients and Careg

Primary Outcomes measured in trial *

comma-separated list of primary outcomes reported in the trial

Alzheimer’s Disease Assessment Scale-cogniti

Secondary/other outcomes

Are there any other outcomes the intervention is expected to affect?

Alzheimer’s Disease Assessment Scale-cognitive subscale 14, Korean Mini Mental State Examination, Clinical Dementia Rating – Sum of Boxes, Alzheimer's Disease Composite Score, Digit Symbol Coding, Clinician Interview Based Impression of Severity and Clinician Interview Based Impression of Change plus Caregiver Input, Alzheimer’s Disease Cooperative Study - Mild Cognitive Impairment - Activities of Daily Living, EuroQol-5 Dimensions-3 Level and EuroQol-5 Dimensions-Visual Analogue Scale

Recommended "Dose" *

What do the instructions for users say on how often the app should be used?

- (●) Approximately Daily
- ( ) Approximately Weekly
- ( ) Approximately Monthly
- ( ) Approximately Yearly
- ( ) "as needed"
- ( ) 기타:

Approx. Percentage of Users (starters) still using the app as recommended after 3 months *

- ( ) unknown / not evaluated
- ( ) 0-10%
- ( ) 11-20%
- ( ) 21-30%
- ( ) 31-40%
- ( ) 41-50%
- ( ) 51-60%
- ( ) 61-70%
- (●) 71%-80%
- ( ) 81-90%
- ( ) 91-100%
- ( ) 기타:

Overall, was the app/intervention effective? *

- ( ) yes: all primary outcomes were significantly better in intervention group vs control
- (•) partly: SOME primary outcomes were significantly better in intervention group vs control
- ( ) no statistically significant difference between control and intervention
- ( ) potentially harmful: control was significantly better than intervention in one or more outcomes
- ( ) inconclusive: more research is needed
- ( ) 기타:

Article Preparation Status/Stage *

At which stage in your article preparation are you currently (at the time you fill in this form)

- ( ) not submitted yet - in early draft status
- (•) not submitted yet - in late draft status, just before submission
- ( ) submitted to a journal but not reviewed yet
- ( ) submitted to a journal and after receiving initial reviewer comments
- ( ) submitted to a journal and accepted, but not published yet
- ( ) published
- ( ) 기타:

Journal *

If you already know where you will submit this paper (or if it is already submitted), please provide the journal name (if it is not JMIR, provide the journal name under "other")

- ○ not submitted yet / unclear where I will submit this
- ◉ Journal of Medical Internet Research (JMIR)
- ○ JMIR mHealth and UHealth
- ○ JMIR Serious Games
- ○ JMIR Mental Health
- ○ JMIR Public Health
- ○ JMIR Formative Research
- ○ Other JMIR sister journal
- ○ 기타:

Is this a full powered effectiveness trial or a pilot/feasibility trial? *

- ○ Pilot/feasibility
- ◉ Fully powered

Manuscript tracking number *

If this is a JMIR submission, please provide the manuscript tracking number under "other" (The ms tracking number can be found in the submission acknowledgement email, or when you login as author in JMIR. If the paper is already published in JMIR, then the ms tracking number is the four-digit number at the end of the DOI, to be found at the bottom of each published article in JMIR)

- ◉ no ms number (yet) / not (yet) submitted to / published in JMIR
- ○ 기타:

TITLE AND ABSTRACT

1a) TITLE: Identification as a randomized trial in the title

1a) Does your paper address CONSORT item 1a? *

I.e does the title contain the phrase "Randomized Controlled Trial"? (if not, explain the reason under "other")

- (selected) yes
- ( ) 기타:

1a-i) Identify the mode of delivery in the title

Identify the mode of delivery. Preferably use "web-based" and/or "mobile" and/or "electronic game" in the title. Avoid ambiguous terms like "online", "virtual", "interactive". Use "Internet-based" only if Intervention includes non-web-based Internet components (e.g. email), use "computer-based" or "electronic" only if offline products are used. Use "virtual" only in the context of "virtual reality" (3-D worlds). Use "online" only in the context of "online support groups". Complement or substitute product names with broader terms for the class of products (such as "mobile" or "smart phone" instead of "iphone"), especially if the application runs on different platforms.

	1	2	3	4	5	
subitem not at all important	( )	( )	( )	( )	(selected)	essential

선택해제

Does your paper address subitem 1a-i? *

Copy and paste relevant sections from manuscript title (include quotes in quotation marks "like this" to indicate direct quotes from your manuscript), or elaborate on this item by providing additional information not in the ms, or briefly explain why the item is not applicable/relevant for your study

The Efficacy and Safety of a "Mobile App-Based" Metamemory Cognitive Training for Mild Cognitive Impairment: Multicenter, Randomized Clinical Trial

1a-ii) Non-web-based components or important co-interventions in title

Mention non-web-based components or important co-interventions in title, if any (e.g., “with telephone support”).

	1	2	3	4	5	
subitem not at all important	◉	○	○	○	○	essential

선택해제

Does your paper address subitem 1a-ii?

Copy and paste relevant sections from manuscript title (include quotes in quotation marks "like this" to indicate direct quotes from your manuscript), or elaborate on this item by providing additional information not in the ms, or briefly explain why the item is not applicable/relevant for your study

We used a mobile app-based modality for both the intervention group and the sham device control group, and a non-web-based component was not used.

1a-iii) Primary condition or target group in the title

Mention primary condition or target group in the title, if any (e.g., “for children with Type I Diabetes”) Example: A Web-based and Mobile Intervention with Telephone Support for Children with Type I Diabetes: Randomized Controlled Trial

	1	2	3	4	5	
subitem not at all important	○	○	○	○	◉	essential

선택해제

Does your paper address subitem 1a-iii? *

Copy and paste relevant sections from manuscript title (include quotes in quotation marks "like this" to indicate direct quotes from your manuscript), or elaborate on this item by providing additional information not in the ms, or briefly explain why the item is not applicable/relevant for your study

The Efficacy and Safety of a Mobile App-Based Metamemory Cognitive Training for "Mild Cognitive Impairment": Multicenter, Randomized Clinical Trial

1b) ABSTRACT: Structured summary of trial design, methods, results, and conclusions

NPT extension: Description of experimental treatment, comparator, care providers, centers, and blinding status.

1b-i) Key features/functionalities/components of the intervention and comparator in the METHODS section of the ABSTRACT

Mention key features/functionalities/components of the intervention and comparator in the abstract. If possible, also mention theories and principles used for designing the site. Keep in mind the needs of systematic reviewers and indexers by including important synonyms. (Note: Only report in the abstract what the main paper is reporting. If this information is missing from the main body of text, consider adding it)

	1	2	3	4	5	
subitem not at all important	○	○	○	◉	○	essential

선택해제

Does your paper address subitem 1b-i? *

Copy and paste relevant sections from the manuscript abstract (include quotes in quotation marks "like this" to indicate direct quotes from your manuscript), or elaborate on this item by providing additional information not in the ms, or briefly explain why the item is not applicable/relevant for your study

We aimed to evaluate the efficacy and safety of the "mobile app-based MMT, ET-101", compared to a "sham device control group" in individuals with MCI. "The intervention lasted 12 weeks, with a 12-week follow-up". The ET-101 group received "metamemory-based multi-memory strategy training and real-time feedback".

1b-ii) Level of human involvement in the METHODS section of the ABSTRACT

Clarify the level of human involvement in the abstract, e.g., use phrases like “fully automated” vs. “therapist/nurse/care provider/physician-assisted” (mention number and expertise of providers involved, if any). (Note: Only report in the abstract what the main paper is reporting. If this information is missing from the main body of text, consider adding it)

	1	2	3	4	5	
subitem not at all important	(•)	○	○	○	○	essential

선택해제

Does your paper address subitem 1b-ii?

Copy and paste relevant sections from the manuscript abstract (include quotes in quotation marks "like this" to indicate direct quotes from your manuscript), or elaborate on this item by providing additional information not in the ms, or briefly explain why the item is not applicable/relevant for your study

In this study, both the training group and the sham device control group used only a mobile app, with no other human involvement in the training process.

1b-iii) Open vs. closed, web-based (self-assessment) vs. face-to-face assessments in the METHODS section of the ABSTRACT

Mention how participants were recruited (online vs. offline), e.g., from an open access website or from a clinic or a closed online user group (closed usergroup trial), and clarify if this was a purely web-based trial, or there were face-to-face components (as part of the intervention or for assessment). Clearly say if outcomes were self-assessed through questionnaires (as common in web-based trials). Note: In traditional offline trials, an open trial (open-label trial) is a type of clinical trial in which both the researchers and participants know which treatment is being administered. To avoid confusion, use “blinded” or “unblinded” to indicated the level of blinding instead of “open”, as “open” in web-based trials usually refers to “open access” (i.e. participants can self-enrol). (Note: Only report in the abstract what the main paper is reporting. If this information is missing from the main body of text, consider adding it)

	1	2	3	4	5	
subitem not at all important	○	○	○	◉	○	essential

선택해제

Does your paper address subitem 1b-iii?

Copy and paste relevant sections from the manuscript abstract (include quotes in quotation marks "like this" to indicate direct quotes from your manuscript), or elaborate on this item by providing additional information not in the ms, or briefly explain why the item is not applicable/relevant for your study

This multicenter, randomized controlled trial enrolled MCI participants, recruited from "seven medical centers", and randomly assigned them to the ET-101 or control group (1:1 ratio).

1b-iv) RESULTS section in abstract must contain use data

Report number of participants enrolled/assessed in each group, the use/uptake of the intervention (e.g., attrition/adherence metrics, use over time, number of logins etc.), in addition to primary/secondary outcomes. (Note: Only report in the abstract what the main paper is reporting. If this information is missing from the main body of text, consider adding it)

	1	2	3	4	5	
subitem not at all important	○	○	○	◉	○	essential

선택해제

Does your paper address subitem 1b-iv?

Copy and paste relevant sections from the manuscript abstract (include quotes in quotation marks "like this" to indicate direct quotes from your manuscript), or elaborate on this item by providing additional information not in the ms, or briefly explain why the item is not applicable/relevant for your study

In the full analysis set, "49 participants were included in the ET-101 group and 50" in the control group. "The attrition rate in the ET-101 was 22.4%".

1b-v) CONCLUSIONS/DISCUSSION in abstract for negative trials

Conclusions/Discussions in abstract for negative trials: Discuss the primary outcome - if the trial is negative (primary outcome not changed), and the intervention was not used, discuss whether negative results are attributable to lack of uptake and discuss reasons. (Note: Only report in the abstract what the main paper is reporting. If this information is missing from the main body of text, consider adding it)

	1	2	3	4	5	
subitem not at all important	○	◉	○	○	○	essential

선택해제

Does your paper address subitem 1b-v?

Copy and paste relevant sections from the manuscript abstract (include quotes in quotation marks "like this" to indicate direct quotes from your manuscript), or elaborate on this item by providing additional information not in the ms, or briefly explain why the item is not applicable/relevant for your study

At week 24, "the proportion of responders who maintained or improved their ADAS-Cog-14 scores was significantly higher in the ET-101 group than in the control group (P = .002)". Additionally, "the ET-101 group showed a significant improvement in ADAS-Cog-14 scores at week 24 compared to baseline (estimates: -2.53, t_{65} = 3.05, Bonferroni-adjusted P = .003)".

INTRODUCTION

2a) In INTRODUCTION: Scientific background and explanation of rationale

2a-i) Problem and the type of system/solution

Describe the problem and the type of system/solution that is object of the study: intended as stand-alone intervention vs. incorporated in broader health care program? Intended for a particular patient population? Goals of the intervention, e.g., being more cost-effective to other interventions, replace or complement other solutions? (Note: Details about the intervention are provided in “Methods” under 5)

	1	2	3	4	5	
subitem not at all important	○	○	○	◉	○	essential

선택해제

Does your paper address subitem 2a-i? *

Copy and paste relevant sections from the manuscript (include quotes in quotation marks "like this" to indicate direct quotes from your manuscript), or elaborate on this item by providing additional information not in the ms, or briefly explain why the item is not applicable/relevant for your study

Conventionally, the main limitation of cognitive interventions for "MCI" is their reliance on "face-to-face interactions with professionals, which hampers the accessibility and continuity of training". However, "digitalization is ideal for facilitating the migration of care from clinics to patients' homes, thereby overcoming constraints related to time and space".

2a-ii) Scientific background, rationale: What is known about the (type of) system

Scientific background, rationale: What is known about the (type of) system that is the object of the study (be sure to discuss the use of similar systems for other conditions/diagnoses, if appropiate), motivation for the study, i.e. what are the reasons for and what is the context for this specific study, from which stakeholder viewpoint is the study performed, potential impact of findings [2]. Briefly justify the choice of the comparator.

	1	2	3	4	5	
subitem not at all important	○	○	○	○	◉	essential

선택해제

Does your paper address subitem 2a-ii? *

Copy and paste relevant sections from the manuscript (include quotes in quotation marks "like this" to indicate direct quotes from your manuscript), or elaborate on this item by providing additional information not in the ms, or briefly explain why the item is not applicable/relevant for your study

Study group participants underwent ET-101, a mobile application-based "MMT program incorporating multi-memory strategies". ET-101 is based on a "MMT program developed by Youn and colleagues" and was produced by Emocog Inc. "We previously introduced the offline and smart-speaker-based versions of this MMT program, and their effectiveness has been well-validated".

2b) In INTRODUCTION: Specific objectives or hypotheses

Does your paper address CONSORT subitem 2b? *

Copy and paste relevant sections from the manuscript (include quotes in quotation marks "like this" to indicate direct quotes from your manuscript), or elaborate on this item by providing additional information not in the ms, or briefly explain why the item is not applicable/relevant for your study

Thus, metamemory-based digital cognitive training can be expected to enhance the benefits of cognitive training in individuals with MCI by addressing the limitations of previous cognitive interventions. Based on this concept, ET-101—a mobile application-based MMT — was developed. In this randomized clinical trial, "we aimed to evaluate the efficacy and safety of ET-101 in elderly individuals with MCI compared to a control group using a sham device."

METHODS

3a) Description of trial design (such as parallel, factorial) including allocation ratio

Does your paper address CONSORT subitem 3a? *

Copy and paste relevant sections from the manuscript (include quotes in quotation marks "like this" to indicate direct quotes from your manuscript), or elaborate on this item by providing additional information not in the ms, or briefly explain why the item is not applicable/relevant for your study

"This was a multicenter, 24-weeks parallel, randomized, sham device-controlled study of ET-101 (Cogthera)"

3b) Important changes to methods after trial commencement (such as eligibility criteria), with reasons

Does your paper address CONSORT subitem 3b? *

Copy and paste relevant sections from the manuscript (include quotes in quotation marks "like this" to indicate direct quotes from your manuscript), or elaborate on this item by providing additional information not in the ms, or briefly explain why the item is not applicable/relevant for your study

We did not have important changes to methods after trial commencement.

3b-i) Bug fixes, Downtimes, Content Changes

Bug fixes, Downtimes, Content Changes: ehealth systems are often dynamic systems. A description of changes to methods therefore also includes important changes made on the intervention or comparator during the trial (e.g., major bug fixes or changes in the functionality or content) (5-iii) and other “unexpected events” that may have influenced study design such as staff changes, system failures/downtimes, etc. [2].

	1	2	3	4	5	
subitem not at all important	◉	○	○	○	○	essential

선택해제

Does your paper address subitem 3b-i?

Copy and paste relevant sections from the manuscript (include quotes in quotation marks "like this" to indicate direct quotes from your manuscript), or elaborate on this item by providing additional information not in the ms, or briefly explain why the item is not applicable/relevant for your study

We did not have bug fixes, downtimes, content changes during the clinical trial

4a) Eligibility criteria for participants

Does your paper address CONSORT subitem 4a? *

Copy and paste relevant sections from the manuscript (include quotes in quotation marks "like this" to indicate direct quotes from your manuscript), or elaborate on this item by providing additional information not in the ms, or briefly explain why the item is not applicable/relevant for your study

Participants who met the following inclusion criteria were included in the study: (1) individuals aged 55 to 85 years; (2) patients diagnosed with amnestic MCI by trained psychiatrists or neurologists according to Petersen's criteria, characterized by cognitive complaints and objective memory impairment determined by either the Consortium to Establish a Registry for Alzheimer's Disease neuropsychological assessment battery (CERAD-NP) or the Seoul Neuropsychological Screening Battery (SNSB) verbal learning test. Memory impairment was defined as a z-score of -1 or lower in at least one of the following subtests: immediate word recall score, delayed word recall score, or delayed word recognition score of verbal learning test in CERAD-NP or SNSB, while maintaining essentially normal functional activities (3)The Mini Mental State Examination (MMSE) score of 27 or lower; (4) not meeting the diagnostic criteria for dementia; (5) a global Clinical Dementia Rating (CDR) score of 0.5; (6) if prescribed cognitive enhancers (Acetylcholinesterase inhibitors or memantine), they must have been on a stable dose for at least 12 weeks prior to randomization; (7) having a caregiver who spends more than 8 hours per week with the participant and agrees to assist with the participant's follow-up and clinical evaluations; (8) possession of a personal smartphone and no difficulties using mobile apps; (9) ability to make phone calls to a caregiver independently using the smartphone; (10) no difficulties reading or writing in Korean; (11) adequate vision and hearing for participating in the clinical trial.

Participants were excluded from the study if any of the following exclusion criteria were met: (1) history of transient ischemic attack, stroke, or seizure within the past 12 month, (2) history of severe psychiatric disorders or currently under unstable psychiatric symptoms, (3) active suicidal intent as assessed by the Columbia-Suicide Severity Rating Scale, or a history of treatment for suicidal behavior within the past 5 years , (4) unstable findings on physical examination, neurological examination, vital signs, or the presence of an ongoing unstable physical illness, (5) substance dependence within the past 2 years, (6) Use of prohibited medications as outlined in the Supplementary 1, (7) scheduled to undergo surgery requiring general anesthesia, (8) participation in any form of cognitive intervention within the past 3 months.

4a-i) Computer / Internet literacy

Computer / Internet literacy is often an implicit “de facto” eligibility criterion - this should be explicitly clarified.

	1	2	3	4	5	
subitem not at all important	○	○	○	○	●	essential

선택해제

Does your paper address subitem 4a-i?

Copy and paste relevant sections from the manuscript (include quotes in quotation marks "like this" to indicate direct quotes from your manuscript), or elaborate on this item by providing additional information not in the ms, or briefly explain why the item is not applicable/relevant for your study

(8) "possession of a personal smartphone and no difficulties using mobile apps"; (9) "ability to make phone calls to a caregiver independently using the smartphone";

4a-ii) Open vs. closed, web-based vs. face-to-face assessments:

Open vs. closed, web-based vs. face-to-face assessments: Mention how participants were recruited (online vs. offline), e.g., from an open access website or from a clinic, and clarify if this was a purely web-based trial, or there were face-to-face components (as part of the intervention or for assessment), i.e., to what degree got the study team to know the participant. In online-only trials, clarify if participants were quasi-anonymous and whether having multiple identities was possible or whether technical or logistical measures (e.g., cookies, email confirmation, phone calls) were used to detect/prevent these.

	1	2	3	4	5	
subitem not at all important	○	○	○	○	●	essential

선택해제

Does your paper address subitem 4a-ii? *

Copy and paste relevant sections from the manuscript (include quotes in quotation marks "like this" to indicate direct quotes from your manuscript), or elaborate on this item by providing additional information not in the ms, or briefly explain why the item is not applicable/relevant for your study

The participants were "recruited through memory clinics of seven medical centers in Republic of Korea". "The scales listed below were assessed by a trained psychologist in face-to-face". (This study utilized a mobile app for training, with no human involvement in the training process. All assessments evaluating the effectiveness of the training were conducted by trained psychologists based on interviews with informants.)

4a-iii) Information giving during recruitment

Information given during recruitment. Specify how participants were briefed for recruitment and in the informed consent procedures (e.g., publish the informed consent documentation as appendix, see also item X26), as this information may have an effect on user self-selection, user expectation and may also bias results.

	1	2	3	4	5	
subitem not at all important	○	○	○	○	◉	essential

선택해제

Does your paper address subitem 4a-iii?

Copy and paste relevant sections from the manuscript (include quotes in quotation marks "like this" to indicate direct quotes from your manuscript), or elaborate on this item by providing additional information not in the ms, or briefly explain why the item is not applicable/relevant for your study

The participants were recruited through memory clinics of "seven medical centers" in Republic of Korea. "All participants provided written, informed consent to participate, and their caregivers were asked to provide separate written, informed consent".

4b) Settings and locations where the data were collected

Does your paper address CONSORT subitem 4b? *

Copy and paste relevant sections from the manuscript (include quotes in quotation marks "like this" to indicate direct quotes from your manuscript), or elaborate on this item by providing additional information not in the ms, or briefly explain why the item is not applicable/relevant for your study

The participants were recruited through "memory clinics of seven medical centers in Republic of Korea". "The participants recruited from June, 2023 to December, 2023".

4b-i) Report if outcomes were (self-)assessed through online questionnaires

Clearly report if outcomes were (self-)assessed through online questionnaires (as common in web-based trials) or otherwise.

	1	2	3	4	5	
subitem not at all important	◉	○	○	○	○	essential

선택해제

Does your paper address subitem 4b-i? *

Copy and paste relevant sections from the manuscript (include quotes in quotation marks "like this" to indicate direct quotes from your manuscript), or elaborate on this item by providing additional information not in the ms, or briefly explain why the item is not applicable/relevant for your study

All assessments presented in the study were conducted face-to-face by trained psychologists with the participants.

4b-ii) Report how institutional affiliations are displayed

Report how institutional affiliations are displayed to potential participants [on ehealth media], as affiliations with prestigious hospitals or universities may affect volunteer rates, use, and reactions with regards to an intervention.(Not a required item – describe only if this may bias results)

	1	2	3	4	5	
subitem not at all important	◉	○	○	○	○	essential

선택해제

Does your paper address subitem 4b-ii?

Copy and paste relevant sections from the manuscript (include quotes in quotation marks "like this" to indicate direct quotes from your manuscript), or elaborate on this item by providing additional information not in the ms, or briefly explain why the item is not applicable/relevant for your study

내 답변

5) The interventions for each group with sufficient details to allow replication, including how and when they were actually administered

5-i) Mention names, credential, affiliations of the developers, sponsors, and owners

Mention names, credential, affiliations of the developers, sponsors, and owners [6] (if authors/evaluators are owners or developer of the software, this needs to be declared in a “Conflict of interest” section or mentioned elsewhere in the manuscript).

	1	2	3	4	5	
subitem not at all important	○	○	○	○	◉	essential

선택해제

Does your paper address subitem 5-i?

Copy and paste relevant sections from the manuscript (include quotes in quotation marks "like this" to indicate direct quotes from your manuscript), or elaborate on this item by providing additional information not in the ms, or briefly explain why the item is not applicable/relevant for your study

ET-101 is based on a MMT program developed by "Youn and colleagues" and was produced by "Emocog Inc."

5-ii) Describe the history/development process

Describe the history/development process of the application and previous formative evaluations (e.g., focus groups, usability testing), as these will have an impact on adoption/use rates and help with interpreting results.

	1	2	3	4	5	
subitem not at all important	○	○	○	◉	○	essential

선택해제

Does your paper address subitem 5-ii?

Copy and paste relevant sections from the manuscript (include quotes in quotation marks "like this" to indicate direct quotes from your manuscript), or elaborate on this item by providing additional information not in the ms, or briefly explain why the item is not applicable/relevant for your study

Study group participants underwent ET-101, a mobile application-based MMT program incorporating multi-memory strategies. ET-101 is based on a "MMT program developed by Youn and colleagues" and was produced by Emocog Inc. "We previously introduced the offline and smart-speaker-based versions of this MMT program, and their effectiveness has been well-validated".

5-iii) Revisions and updating

Revisions and updating. Clearly mention the date and/or version number of the application/intervention (and comparator, if applicable) evaluated, or describe whether the intervention underwent major changes during the evaluation process, or whether the development and/or content was “frozen” during the trial. Describe dynamic components such as news feeds or changing content which may have an impact on the replicability of the intervention (for unexpected events see item 3b).

	1	2	3	4	5	
subitem not at all important	(●)	( )	( )	( )	( )	essential

선택해제

Does your paper address subitem 5-iii?

Copy and paste relevant sections from the manuscript (include quotes in quotation marks "like this" to indicate direct quotes from your manuscript), or elaborate on this item by providing additional information not in the ms, or briefly explain why the item is not applicable/relevant for your study

The ET-101 used in the clinical trial currently exists in only a single version (Release 2023.06.08)

5-iv) Quality assurance methods

Provide information on quality assurance methods to ensure accuracy and quality of information provided [1], if applicable.

	1	2	3	4	5	
subitem not at all important	( )	( )	( )	( )	(●)	essential

선택해제

Does your paper address subitem 5-iv?

Copy and paste relevant sections from the manuscript (include quotes in quotation marks "like this" to indicate direct quotes from your manuscript), or elaborate on this item by providing additional information not in the ms, or briefly explain why the item is not applicable/relevant for your study

"A statistician, independent from the clinical team", carried out the block randomization, assigning participants to either the study group or the control group in a 1:1 ratio. Throughout the trial, "psychiatrists, neurologists, and outcome raters were blinded to treatment assignment".

5-v) Ensure replicability by publishing the source code, and/or providing screenshots/screen-capture video, and/or providing flowcharts of the algorithms used

Ensure replicability by publishing the source code, and/or providing screenshots/screen-capture video, and/or providing flowcharts of the algorithms used. Replicability (i.e., other researchers should in principle be able to replicate the study) is a hallmark of scientific reporting.

	1	2	3	4	5	
subitem not at all important	○	○	○	◉	○	essential

선택해제

Does your paper address subitem 5-v?

Copy and paste relevant sections from the manuscript (include quotes in quotation marks "like this" to indicate direct quotes from your manuscript), or elaborate on this item by providing additional information not in the ms, or briefly explain why the item is not applicable/relevant for your study

ET-101 includes cognitive exercises specifically designed to incorporate memory strategies focusing on attention, imagination, and associations. According to the memory encoding hypothesis and neuroscience findings, these strategies have been shown to effectively address issues with memory encoding and retrieval commonly observed in the elderly with cognitive decline[22, 23]. Furthermore, they are known to strengthen related brain regions, including the prefrontal cortex, hippocampus, parahippocampus, precuneus, and the cingulum that connects these areas[22, 24]. Attention training includes exercises that enhance selective focus on stimuli to be memorized, as well as tasks aimed at improving processing speed and increasing working memory. Imagination training facilitates the transition to long-term memory by linking spatial-temporal memory and visual imagery associated with the objects to be memorized. In association training, two or more pieces of information that need to be remembered are connected and assigned mutual meaning, thereby strengthening semantic cue to aid long-term memory formation. The detailed content of each activity can be found in the supplementary 2.
The study group engaged in a 12-week training program using ET-101. The program was structured to involve two sessions each day, seven days a week, lasting approximately 15minutes per session. The initial session includes three representative cognitive exercises, one each for attention, imagination, and association. In the later session, four additional exercises are conducted, selected from nine available options, excluding the three exercises from the initial session, based on a personalized algorithm. A push alarm was provided daily to encourage training.
(Details of the training will be provided in the supplementary file)

5-vi) Digital preservation

Digital preservation: Provide the URL of the application, but as the intervention is likely to change or disappear over the course of the years; also make sure the intervention is archived (Internet Archive, webcitation.org, and/or publishing the source code or screenshots/videos alongside the article). As pages behind login screens cannot be archived, consider creating demo pages which are accessible without login.

	1	2	3	4	5	
subitem not at all important	◉	○	○	○	○	essential

선택해제

Does your paper address subitem 5-vi?

Copy and paste relevant sections from the manuscript (include quotes in quotation marks "like this" to indicate direct quotes from your manuscript), or elaborate on this item by providing additional information not in the ms, or briefly explain why the item is not applicable/relevant for your study

We do not have the open URL yet.

5-vii) Access

Access: Describe how participants accessed the application, in what setting/context, if they had to pay (or were paid) or not, whether they had to be a member of specific group. If known, describe how participants obtained “access to the platform and Internet” [1]. To ensure access for editors/reviewers/readers, consider to provide a “backdoor” login account or demo mode for reviewers/readers to explore the application (also important for archiving purposes, see vi).

	1	2	3	4	5	
subitem not at all important	◉	○	○	○	○	essential

선택해제

Does your paper address subitem 5-vii? *

Copy and paste relevant sections from the manuscript (include quotes in quotation marks "like this" to indicate direct quotes from your manuscript), or elaborate on this item by providing additional information not in the ms, or briefly explain why the item is not applicable/relevant for your study

It is not paid, but used just for the study.

5-viii) Mode of delivery, features/functionalities/components of the intervention and comparator, and the theoretical framework

Describe mode of delivery, features/functionalities/components of the intervention and comparator, and the theoretical framework [6] used to design them (instructional strategy [1], behaviour change techniques, persuasive features, etc., see e.g., [7, 8] for terminology). This includes an in-depth description of the content (including where it is coming from and who developed it) [1],” whether [and how] it is tailored to individual circumstances and allows users to track their progress and receive feedback” [6]. This also includes a description of communication delivery channels and – if computer-mediated communication is a component – whether communication was synchronous or asynchronous [6]. It also includes information on presentation strategies [1], including page design principles, average amount of text on pages, presence of hyperlinks to other resources, etc. [1].

	1	2	3	4	5	
subitem not at all important	○	○	○	○	◉	essential

선택해제

Does your paper address subitem 5-viii? *

Copy and paste relevant sections from the manuscript (include quotes in quotation marks "like this" to indicate direct quotes from your manuscript), or elaborate on this item by providing additional information not in the ms, or briefly explain why the item is not applicable/relevant for your study

Study group participants underwent ET-101, a "mobile application-based MMT program" incorporating multi-memory strategies. Real-time metamemory teaching is a primary feature of ET-101. The training agent of ET-101 interacts with users through voice-based, two-way communication to enhance metamemory, including metaknowledge, meta-monitoring, and meta-judgment. Regarding metaknowledge, the agent begins the training by asking users questions about their memory encoding related to long-term memory and the patterns of memory strategies they currently use in daily life. Additionally, during training sessions, the agent introduces multi-memory strategies that can enhance long-term memory and explains how these strategies can improve memory performance. This helps users gain objective insight into their cognitive levels and understand how to compensate for cognitive deficits. Regarding meta-monitoring and meta-judgment, the agent explains the daily trends in users' memory span based on their training results. The agent identifies users' strong and weak memory strategies, helping them recognize their cognitive strengths and weaknesses. Additionally, at the end of the training, the agent provides guidance on how to apply the learned strategies to daily life, facilitating the generalization of memory strategy use. Throughout the entire metamemory process, the agent provides personalized feedback, adjusts difficulty levels, and modifies training tasks based on users' responses and training results.

ET-101 includes cognitive exercises specifically designed to incorporate memory strategies focusing on attention, imagination, and associations. According to the memory encoding hypothesis and neuroscience findings, these strategies have been shown to effectively address issues with memory encoding and retrieval commonly observed in the elderly with cognitive decline[22, 23]. Furthermore, they are known to strengthen related brain regions, including the prefrontal cortex, hippocampus, parahippocampus, precuneus, and the cingulum that connects these areas[22, 24]. Attention training includes exercises that enhance selective focus on stimuli to be memorized, as well as tasks aimed at improving processing speed and increasing working memory. Imagination training facilitates the transition to long-term memory by linking spatial-temporal memory and visual imagery associated with the objects to be memorized. In association training, two or more pieces of information that need to be remembered are connected and assigned mutual meaning, thereby strengthening semantic cue to aid long-term memory formation. The detailed content of each activity can be found in the supplementary 2.

5-ix) Describe use parameters

Describe use parameters (e.g., intended “doses” and optimal timing for use). Clarify what instructions or recommendations were given to the user, e.g., regarding timing, frequency, heaviness of use, if any, or was the intervention used ad libitum.

	1	2	3	4	5	
subitem not at all important	○	○	○	●	○	essential

선택해제

Does your paper address subitem 5-ix?

Copy and paste relevant sections from the manuscript (include quotes in quotation marks "like this" to indicate direct quotes from your manuscript), or elaborate on this item by providing additional information not in the ms, or briefly explain why the item is not applicable/relevant for your study

The study group engaged in a 12-week training program using ET-101. The program was structured to involve "two sessions each day, seven days a week, lasting approximately 15minutes per session".

5-x) Clarify the level of human involvement

Clarify the level of human involvement (care providers or health professionals, also technical assistance) in the e-intervention or as co-intervention (detail number and expertise of professionals involved, if any, as well as “type of assistance offered, the timing and frequency of the support, how it is initiated, and the medium by which the assistance is delivered”. It may be necessary to distinguish between the level of human involvement required for the trial, and the level of human involvement required for a routine application outside of a RCT setting (discuss under item 21 – generalizability).

	1	2	3	4	5	
subitem not at all important	●	○	○	○	○	essential

선택해제

Does your paper address subitem 5-x?

Copy and paste relevant sections from the manuscript (include quotes in quotation marks "like this" to indicate direct quotes from your manuscript), or elaborate on this item by providing additional information not in the ms, or briefly explain why the item is not applicable/relevant for your study

In this clinical trial, the training process involved no human involvement.

5-xi) Report any prompts/reminders used

Report any prompts/reminders used: Clarify if there were prompts (letters, emails, phone calls, SMS) to use the application, what triggered them, frequency etc. It may be necessary to distinguish between the level of prompts/reminders required for the trial, and the level of prompts/reminders for a routine application outside of a RCT setting (discuss under item 21 – generalizability).

	1	2	3	4	5	
subitem not at all important	○	○	○	○	◉	essential

선택해제

Does your paper address subitem 5-xi? *

Copy and paste relevant sections from the manuscript (include quotes in quotation marks "like this" to indicate direct quotes from your manuscript), or elaborate on this item by providing additional information not in the ms, or briefly explain why the item is not applicable/relevant for your study

A push alarm was provided daily to encourage training.

5-xii) Describe any co-interventions (incl. training/support)

Describe any co-interventions (incl. training/support): Clearly state any interventions that are provided in addition to the targeted eHealth intervention, as ehealth intervention may not be designed as stand-alone intervention. This includes training sessions and support [1]. It may be necessary to distinguish between the level of training required for the trial, and the level of training for a routine application outside of a RCT setting (discuss under item 21 – generalizability.

	1	2	3	4	5	
subitem not at all important	◉	○	○	○	○	essential

선택해제

Does your paper address subitem 5-xii? *

Copy and paste relevant sections from the manuscript (include quotes in quotation marks "like this" to indicate direct quotes from your manuscript), or elaborate on this item by providing additional information not in the ms, or briefly explain why the item is not applicable/relevant for your study

The application was stand-alone intervention not using any other interventions.

6a) Completely defined pre-specified primary and secondary outcome measures, including how and when they were assessed

Does your paper address CONSORT subitem 6a? *

Copy and paste relevant sections from the manuscript (include quotes in quotation marks "like this" to indicate direct quotes from your manuscript), or elaborate on this item by providing additional information not in the ms, or briefly explain why the item is not applicable/relevant for your study

These clinical measures and outcomes were prespecified in ClinicalTrials.gov (NCT05938426).

6a-i) Online questionnaires: describe if they were validated for online use and apply CHERRIES items to describe how the questionnaires were designed/deployed

If outcomes were obtained through online questionnaires, describe if they were validated for online use and apply CHERRIES items to describe how the questionnaires were designed/deployed [9].

	1	2	3	4	5	
subitem not at all important	◉	○	○	○	○	essential

선택해제

Does your paper address subitem 6a-i?

Copy and paste relevant sections from manuscript text

The clinical measures presented in this study were not obtained through online questionnaires.

6a-ii) Describe whether and how “use” (including intensity of use/dosage) was defined/measured/monitored

Describe whether and how “use” (including intensity of use/dosage) was defined/measured/monitored (logins, logfile analysis, etc.). Use/adoption metrics are important process outcomes that should be reported in any ehealth trial.

	1	2	3	4	5	
subitem not at all important	○	○	○	○	◉	essential

선택해제

Does your paper address subitem 6a-ii?

Copy and paste relevant sections from manuscript text

A training session was considered "completed" only if the "participant finished the entire session for the day".

6a-iii) Describe whether, how, and when qualitative feedback from participants was obtained

Describe whether, how, and when qualitative feedback from participants was obtained (e.g., through emails, feedback forms, interviews, focus groups).

	1	2	3	4	5	
subitem not at all important	○	○	○	○	◉	essential

선택해제

Does your paper address subitem 6a-iii?

Copy and paste relevant sections from manuscript text

The intervention and control groups adhered to an identical visit schedule. Participants completed 5 visits (0-4): Screening (visit 0) included screening for study eligibility; week 0 (visit1, baseline) included baseline demographic data collection and clinical assessments including cognition, function and quality of life, along with randomization; week 6 (visit 2) investigated compliance with study protocol and the occurrence of adverse events; week 12 (visit 3, at the end of the 12-week treatment period) reassessed the same variables from visit 1 and visit 2; week 24 (visit 4, follow-up assessment 12 weeks after treatment completion) reassessed the same variables from visit 3.

6b) Any changes to trial outcomes after the trial commenced, with reasons

Does your paper address CONSORT subitem 6b? *

Copy and paste relevant sections from the manuscript (include quotes in quotation marks "like this" to indicate direct quotes from your manuscript), or elaborate on this item by providing additional information not in the ms, or briefly explain why the item is not applicable/relevant for your study

There was no changes to trial outcomes after the trial commenced.

7a) How sample size was determined

NPT: When applicable, details of whether and how the clustering by care provides or centers was addressed

7a-i) Describe whether and how expected attrition was taken into account when calculating the sample size

Describe whether and how expected attrition was taken into account when calculating the sample size.

	1	2	3	4	5	
subitem not at all important	○	○	○	○	◉	essential

선택해제

Does your paper address subitem 7a-i?

Copy and paste relevant sections from manuscript title (include quotes in quotation marks "like this" to indicate direct quotes from your manuscript), or elaborate on this item by providing additional information not in the ms, or briefly explain why the item is not applicable/relevant for your study

Sample size was calculated based on a two-sample proportion test. The delayed recall has been shown to have the highest predictive accuracy (95.2%) in differentiating dementia from MCI[20]. In a previous study, the proportion of participants showing improvement in delayed recall was 82.76% in the intervention group and 45.16% in the control group[10]. With a two-sided alpha of 0.05 and power of 0.90, and assuming a 1:1 allocation ratio, the minimum sample size per group was determined to be 32 participants. Anticipating a dropout rate of 35%, we intended to enroll 50 participants per group.

7b) When applicable, explanation of any interim analyses and stopping guidelines

Does your paper address CONSORT subitem 7b? *

Copy and paste relevant sections from the manuscript (include quotes in quotation marks "like this" to indicate direct quotes from your manuscript), or elaborate on this item by providing additional information not in the ms, or briefly explain why the item is not applicable/relevant for your study

This study did not conduct an interim analysis. For safety assessment, adverse events were collected weekly according to the CONSORT Harm guideline. In cases where a significant adverse event related to the device used in this study was reported, usage was discontinued.

8a) Method used to generate the random allocation sequence
NPT: When applicable, how care providers were allocated to each trial group

Does your paper address CONSORT subitem 8a? *

Copy and paste relevant sections from the manuscript (include quotes in quotation marks "like this" to indicate direct quotes from your manuscript), or elaborate on this item by providing additional information not in the ms, or briefly explain why the item is not applicable/relevant for your study

"A statistician, independent from the clinical team, carried out the block randomization, assigning participants to either the study group or the control group in a 1:1 ratio".

8b) Type of randomisation; details of any restriction (such as blocking and block size)

Does your paper address CONSORT subitem 8b? *

Copy and paste relevant sections from the manuscript (include quotes in quotation marks "like this" to indicate direct quotes from your manuscript), or elaborate on this item by providing additional information not in the ms, or briefly explain why the item is not applicable/relevant for your study

A statistician, independent from the clinical team, carried out the "block randomization", assigning participants to either the study group or the control group in a 1:1 ratio.

9) Mechanism used to implement the random allocation sequence (such as sequentially numbered containers), describing any steps taken to conceal the sequence until interventions were assigned

Does your paper address CONSORT subitem 9? *

Copy and paste relevant sections from the manuscript (include quotes in quotation marks "like this" to indicate direct quotes from your manuscript), or elaborate on this item by providing additional information not in the ms, or briefly explain why the item is not applicable/relevant for your study

"A statistician, independent from the clinical team, carried out the block randomization, assigning participants to either the study group or the control group in a 1:1 ratio".

10) Who generated the random allocation sequence, who enrolled participants, and who assigned participants to interventions

Does your paper address CONSORT subitem 10? *

Copy and paste relevant sections from the manuscript (include quotes in quotation marks "like this" to indicate direct quotes from your manuscript), or elaborate on this item by providing additional information not in the ms, or briefly explain why the item is not applicable/relevant for your study

"A statistician, independent from the clinical team", carried out the block randomization, assigning participants to either the study group or the control group in a 1:1 ratio. The participants were recruited through memory clinics of seven medical centers in Republic of Korea. The participants recruited from June, 2023 to December, 2023. Participants who met the following inclusion criteria were included in the study (This study included individuals who met the criteria for amnestic MCI based on the Peterson criteria. The determination of whether participants met these criteria was conducted by psychiatrists and neurologists working in memory clinics at seven medical centers.)

11a) If done, who was blinded after assignment to interventions (for example, participants, care providers, those assessing outcomes) and how

NPT: Whether or not administering co-interventions were blinded to group assignment

11a-i) Specify who was blinded, and who wasn’t

Specify who was blinded, and who wasn’t. Usually, in web-based trials it is not possible to blind the participants [1, 3] (this should be clearly acknowledged), but it may be possible to blind outcome assessors, those doing data analysis or those administering co-interventions (if any).

	1	2	3	4	5	
subitem not at all important	○	○	○	○	◉	essential

선택해제

Does your paper address subitem 11a-i? *

Copy and paste relevant sections from the manuscript (include quotes in quotation marks "like this" to indicate direct quotes from your manuscript), or elaborate on this item by providing additional information not in the ms, or briefly explain why the item is not applicable/relevant for your study

Throughout the trial, "psychiatrists, neurologists, and outcome raters" were blinded to treatment assignment.

11a-ii) Discuss e.g., whether participants knew which intervention was the “intervention of interest” and which one was the “comparator”

Informed consent procedures (4a-ii) can create biases and certain expectations - discuss e.g., whether participants knew which intervention was the “intervention of interest” and which one was the “comparator”.

	1	2	3	4	5	
subitem not at all important	○	○	○	◉	○	essential

선택해제

Does your paper address subitem 11a-ii?

Copy and paste relevant sections from the manuscript (include quotes in quotation marks "like this" to indicate direct quotes from your manuscript), or elaborate on this item by providing additional information not in the ms, or briefly explain why the item is not applicable/relevant for your study

Participants were not informed whether they were in the intervention group or the sham device group

11b) If relevant, description of the similarity of interventions

(this item is usually not relevant for ehealth trials as it refers to similarity of a placebo or sham intervention to a active medication/intervention)

Does your paper address CONSORT subitem 11b? *

Copy and paste relevant sections from the manuscript (include quotes in quotation marks "like this" to indicate direct quotes from your manuscript), or elaborate on this item by providing additional information not in the ms, or briefly explain why the item is not applicable/relevant for your study

Training group: The study group engaged in a 12-week training program using ET-101. The program was structured to involve two sessions each day, seven days a week, lasting approximately 15minutes per session. The initial session includes three representative cognitive exercises, one each for attention, imagination, and association. In the later session, four additional exercises are conducted, selected from nine available options, excluding the three exercises from the initial session, based on a personalized algorithm. A push alarm was provided daily to encourage training. A training session was considered completed only if the participant finished the entire session for the day/ Control group: The control group used a sham device over a 12-week period, which featured cognitive assessments and gamification elements but did not include any cognitive training. The guidelines for program usage frequency and reminders was identical to those followed by the study group.

12a) Statistical methods used to compare groups for primary and secondary outcomes

NPT: When applicable, details of whether and how the clustering by care providers or centers was addressed

Does your paper address CONSORT subitem 12a? *

Copy and paste relevant sections from the manuscript (include quotes in quotation marks "like this" to indicate direct quotes from your manuscript), or elaborate on this item by providing additional information not in the ms, or briefly explain why the item is not applicable/relevant for your study

The primary outcome, the proportion of responders and non-responders between the ET-101 and the control group, was compared using the "chi-square test". Secondary outcomes, including clinical measures of cognition, ADL, and quality of life, were assessed by analyzing the changes from baseline to week 12 and week 24. "Independent t-test"s were used to evaluate whether the score changes differed significantly between the two groups. Additionally, a "linear mixed model" analysis was conducted to examine the treatment-time interaction for each clinical assessment.

12a-i) Imputation techniques to deal with attrition / missing values

Imputation techniques to deal with attrition / missing values: Not all participants will use the intervention/comparator as intended and attrition is typically high in ehealth trials. Specify how participants who did not use the application or dropped out from the trial were treated in the statistical analysis (a complete case analysis is strongly discouraged, and simple imputation techniques such as LOCF may also be problematic [4]).

	1	2	3	4	5	
subitem not at all important	○	○	○	○	◉	essential

선택해제

Does your paper address subitem 12a-i? *

Copy and paste relevant sections from the manuscript (include quotes in quotation marks "like this" to indicate direct quotes from your manuscript), or elaborate on this item by providing additional information not in the ms, or briefly explain why the item is not applicable/relevant for your study

Primary outcome: In this study, "non-responder imputation (NRI)" was applied, classifying trial dropouts as non-responders. NRI is a method of handling missing data by assigning all missing cases as non-responders, thereby preventing an overestimation of treatment benefits. This approach is widely recognized and has been extensively adopted in clinical trials investigating drug efficacy across various areas / Secondary analysis: Missing data in clinical scales due to trial dropouts were replaced using "conditional mean imputation using regression analysis". For regression analysis used in imputation, the ADAS-Cog-14, K-MMSE-II, and CDR-SB scores at baseline, as well as the visit and visit-specific values of each clinical measure, were considered. Conditional mean imputation using regression analysis was performed using Python 3.9.20.

12b) Methods for additional analyses, such as subgroup analyses and adjusted analyses

Does your paper address CONSORT subitem 12b? *

Copy and paste relevant sections from the manuscript (include quotes in quotation marks "like this" to indicate direct quotes from your manuscript), or elaborate on this item by providing additional information not in the ms, or briefly explain why the item is not applicable/relevant for your study

For the sensitivity analysis, to confirm that the imputation of missing data did not affect the results, the same linear mixed model analysis and post-hoc analysis were performed "without missing value imputation" for variables where a significant treatment-time interaction was found in secondary outcomes. For the exploratory analysis, the linear mixed model was applied to assess which "subdomains of ADAS-Cog- 14" (memory, language, and praxis) showed significant treatment-time interaction. If a significant treatment-time interaction was presented, a post-hoc analysis was conducted to determine the specific time points, where this interaction was significant. For the "safety analysis", independent t-tests were performed for continuous variables, while chi-square tests or Fisher's exact tests were used for categorical variables.

X26) REB/IRB Approval and Ethical Considerations [recommended as subheading under "Methods"] (not a CONSORT item)

X26-i) Comment on ethics committee approval

	1	2	3	4	5	
subitem not at all important	○	○	○	○	◉	essential

선택해제

Does your paper address subitem X26-i?

Copy and paste relevant sections from the manuscript (include quotes in quotation marks "like this" to indicate direct quotes from your manuscript), or elaborate on this item by providing additional information not in the ms, or briefly explain why the item is not applicable/relevant for your study

The study was conducted following the Declaration of Helsinki and "approved by the Institutional Review Board at each of the seven participating medical centers". The collected data were anonymized.

x26-ii) Outline informed consent procedures

Outline informed consent procedures e.g., if consent was obtained offline or online (how? Checkbox, etc.?), and what information was provided (see 4a-ii). See [6] for some items to be included in informed consent documents.

	1	2	3	4	5	
subitem not at all important	○	○	○	○	◉	essential

선택해제

Does your paper address subitem X26-ii?

Copy and paste relevant sections from the manuscript (include quotes in quotation marks "like this" to indicate direct quotes from your manuscript), or elaborate on this item by providing additional information not in the ms, or briefly explain why the item is not applicable/relevant for your study

All participants provided written, informed consent to participate, and their caregivers were asked to provide separate written, informed consent.

X26-iii) Safety and security procedures

Safety and security procedures, incl. privacy considerations, and any steps taken to reduce the likelihood or detection of harm (e.g., education and training, availability of a hotline)

	1	2	3	4	5	
subitem not at all important	○	○	○	○	●	essential

선택해제

Does your paper address subitem X26-iii?

Copy and paste relevant sections from the manuscript (include quotes in quotation marks "like this" to indicate direct quotes from your manuscript), or elaborate on this item by providing additional information not in the ms, or briefly explain why the item is not applicable/relevant for your study

The collected data were anonymized. The occurrence of adverse events (AEs) was monitored actively and systematically, following the Consolidated Standards of Reporting Trials (CONSORT) Harms guideline[33]. "AEs were assessed weekly by investigators through telephone contact". Additionally, participants were allowed to report AEs spontaneously at any time during the trial. AEs that involve death, life-threatening conditions, hospitalization or prolonged hospitalization, persistent or significant disability or functional impairment, congenital anomalies or birth defects, or other medically important conditions were classified as serious adverse events (SAEs). Furthermore, the relationship between these AEs and the digital therapeutic device used in this trial was evaluated using a five-level categorical scale from "Definitely not related" to "Definitely related".

RESULTS

13a) For each group, the numbers of participants who were randomly assigned, received intended treatment, and were analysed for the primary outcome

NPT: The number of care providers or centers performing the intervention in each group and the number of patients treated by each care provider in each center

Does your paper address CONSORT subitem 13a? *

Copy and paste relevant sections from the manuscript (include quotes in quotation marks "like this" to indicate direct quotes from your manuscript), or elaborate on this item by providing additional information not in the ms, or briefly explain why the item is not applicable/relevant for your study

A total of "140 participants were screened, of whom 40 were excluded based on the inclusion and exclusion criteria" (Figure 1). "The remaining 100 participants were randomized in a 1:1 ratio".

13b) For each group, losses and exclusions after randomisation, together with reasons

Does your paper address CONSORT subitem 13b? (NOTE: Preferably, this is shown in a CONSORT flow diagram) *

Copy and paste relevant sections from the manuscript (include quotes in quotation marks "like this" to indicate direct quotes from your manuscript), or elaborate on this item by providing additional information not in the ms, or briefly explain why the item is not applicable/relevant for your study

A total of 140 participants were screened, of whom 40 were excluded based on the inclusion and exclusion criteria (Figure 1). The remaining 100 participants were randomized in a 1:1 ratio. During the clinical trial period, 11 participants (22.4%) in the ET-101 group and 25 participants (50%) in the control group did not complete the study. (The reasons for participant dropout are detailed and presented in Figure 1)

13b-i) Attrition diagram

Strongly recommended: An attrition diagram (e.g., proportion of participants still logging in or using the intervention/comparator in each group plotted over time, similar to a survival curve) or other figures or tables demonstrating usage/dose/engagement.

	1	2	3	4	5	
subitem not at all important	○	○	○	○	◉	essential

선택해제

Does your paper address subitem 13b-i?

Copy and paste relevant sections from the manuscript or cite the figure number if applicable (include quotes in quotation marks "like this" to indicate direct quotes from your manuscript), or elaborate on this item by providing additional information not in the ms, or briefly explain why the item is not applicable/relevant for your study

The dropout rate and reasons for dropout after enrollment are presented in a flowchart in Figure 1.

14a) Dates defining the periods of recruitment and follow-up

Does your paper address CONSORT subitem 14a? *

Copy and paste relevant sections from the manuscript (include quotes in quotation marks "like this" to indicate direct quotes from your manuscript), or elaborate on this item by providing additional information not in the ms, or briefly explain why the item is not applicable/relevant for your study

The participants recruited from "June, 2023 to December, 2023". The intervention and control groups adhered to an identical visit schedule. Participants completed 5 visits (0-4): Screening (visit 0) included screening for study eligibility; week 0 (visit1, baseline) included baseline demographic data collection and clinical assessments including cognition, function and quality of life, along with randomization; week 6 (visit 2) investigated compliance with study protocol and the occurrence of adverse events; week 12 (visit 3, at the end of the 12-week treatment period) reassessed the same variables from visit 1 and visit 2; week 24 (visit 4, follow-up assessment 12 weeks after treatment completion) reassessed the same variables from visit 3.

14a-i) Indicate if critical “secular events” fell into the study period

Indicate if critical “secular events” fell into the study period, e.g., significant changes in Internet resources available or “changes in computer hardware or Internet delivery resources”

	1	2	3	4	5	
subitem not at all important	◉	○	○	○	○	essential

선택해제

Does your paper address subitem 14a-i?

Copy and paste relevant sections from the manuscript (include quotes in quotation marks "like this" to indicate direct quotes from your manuscript), or elaborate on this item by providing additional information not in the ms, or briefly explain why the item is not applicable/relevant for your study

In this study, participants were provided with a smartphone pre-installed with the app, and no changes in online resources, user systems, or network environments occurred during the trial.

14b) Why the trial ended or was stopped (early)

Does your paper address CONSORT subitem 14b? *

Copy and paste relevant sections from the manuscript (include quotes in quotation marks "like this" to indicate direct quotes from your manuscript), or elaborate on this item by providing additional information not in the ms, or briefly explain why the item is not applicable/relevant for your study

This trial was not interrupted or early stopped and was conducted as planned for the full 24-week follow-up period.

15) A table showing baseline demographic and clinical characteristics for each group

NPT: When applicable, a description of care providers (case volume, qualification, expertise, etc.) and centers (volume) in each group

Does your paper address CONSORT subitem 15? *

Copy and paste relevant sections from the manuscript (include quotes in quotation marks "like this" to indicate direct quotes from your manuscript), or elaborate on this item by providing additional information not in the ms, or briefly explain why the item is not applicable/relevant for your study

Table 1. "Baseline characteristics of study participants"

15-i) Report demographics associated with digital divide issues

In ehealth trials it is particularly important to report demographics associated with digital divide issues, such as age, education, gender, social-economic status, computer/Internet/ehealth literacy of the participants, if known.

	1	2	3	4	5	
subitem not at all important	○	○	○	○	◉	essential

선택해제

Does your paper address subitem 15-i? *

Copy and paste relevant sections from the manuscript (include quotes in quotation marks "like this" to indicate direct quotes from your manuscript), or elaborate on this item by providing additional information not in the ms, or briefly explain why the item is not applicable/relevant for your study

In this study, baseline characteristics, including age, sex, years of education, current medical illness, current use of cognitive enhancers, and relevant clinical assessment results, were compared between the ET-101 and control groups in Table 1.

16) For each group, number of participants (denominator) included in each analysis and whether the analysis was by original assigned groups

16-i) Report multiple “denominators” and provide definitions

Report multiple “denominators” and provide definitions: Report N’s (and effect sizes) “across a range of study participation [and use] thresholds” [1], e.g., N exposed, N consented, N used more than x times, N used more than y weeks, N participants “used” the intervention/comparator at specific pre-defined time points of interest (in absolute and relative numbers per group). Always clearly define “use” of the intervention.

	1	2	3	4	5	
subitem not at all important	○	○	○	○	◉	essential

선택해제

Does your paper address subitem 16-i? *

Copy and paste relevant sections from the manuscript (include quotes in quotation marks "like this" to indicate direct quotes from your manuscript), or elaborate on this item by providing additional information not in the ms, or briefly explain why the item is not applicable/relevant for your study

Among the dropouts for ET-101, one participant did not use the digital therapeutic device even once after randomization. Therefore, the analysis was conducted on a total of 99 participants in the FAS. (In this study, the analysis was conducted on the full analysis set, which included participants who used the assigned digital device at least once after enrollment. The full analysis set follows a modified intention-to-treat (mITT) approach)

16-ii) Primary analysis should be intent-to-treat

Primary analysis should be intent-to-treat, secondary analyses could include comparing only "users", with the appropriate caveats that this is no longer a randomized sample (see 18-i).

	1	2	3	4	5	
subitem not at all important	○	○	○	○	◉	essential

선택해제

Does your paper address subitem 16-ii?

Copy and paste relevant sections from the manuscript (include quotes in quotation marks "like this" to indicate direct quotes from your manuscript), or elaborate on this item by providing additional information not in the ms, or briefly explain why the item is not applicable/relevant for your study

Among the dropouts for ET-101, one participant did not use the digital therapeutic device even once after randomization. Therefore, the analysis was conducted on a total of 99 participants in the FAS. (In this study, the analysis was conducted on the full analysis set, which included participants who used the assigned digital device at least once after enrollment. The full analysis set follows a modified intention-to-treat (mITT) approach)

17a) For each primary and secondary outcome, results for each group, and the estimated effect size and its precision (such as 95% confidence interval)

Does your paper address CONSORT subitem 17a? *

Copy and paste relevant sections from the manuscript (include quotes in quotation marks "like this" to indicate direct quotes from your manuscript), or elaborate on this item by providing additional information not in the ms, or briefly explain why the item is not applicable/relevant for your study

All results were evaluated for statistical significance at a p-value threshold of <0.05. In the post hoc analysis, Bonferroni-adjusted p-values were reported to reduce Type I error. Additionally, F and t scores were provided according to the respective statistical tests.

17a-i) Presentation of process outcomes such as metrics of use and intensity of use

In addition to primary/secondary (clinical) outcomes, the presentation of process outcomes such as metrics of use and intensity of use (dose, exposure) and their operational definitions is critical. This does not only refer to metrics of attrition (13-b) (often a binary variable), but also to more continuous exposure metrics such as “average session length”. These must be accompanied by a technical description how a metric like a “session” is defined (e.g., timeout after idle time) [1] (report under item 6a).

	1	2	3	4	5	
subitem not at all important	O	O	O	O	O	essential

Does your paper address subitem 17a-i?

Copy and paste relevant sections from the manuscript (include quotes in quotation marks "like this" to indicate direct quotes from your manuscript), or elaborate on this item by providing additional information not in the ms, or briefly explain why the item is not applicable/relevant for your study

A training session was considered completed only if the participant finished the entire session for the day. The remaining 100 participants were randomized in a 1:1 ratio. During the clinical trial period, 11 participants (22.4%) in the ET-101 group and 25 participants (50%) in the control group did not complete the study.

17b) For binary outcomes, presentation of both absolute and relative effect sizes is recommended

Does your paper address CONSORT subitem 17b? *

Copy and paste relevant sections from the manuscript (include quotes in quotation marks "like this" to indicate direct quotes from your manuscript), or elaborate on this item by providing additional information not in the ms, or briefly explain why the item is not applicable/relevant for your study

In this study, binary outcomes were presented as incidence rates for each group, and chi-square tests were performed for statistical analysis

18) Results of any other analyses performed, including subgroup analyses and adjusted analyses, distinguishing pre-specified from exploratory

Does your paper address CONSORT subitem 18? *

Copy and paste relevant sections from the manuscript (include quotes in quotation marks "like this" to indicate direct quotes from your manuscript), or elaborate on this item by providing additional information not in the ms, or briefly explain why the item is not applicable/relevant for your study

Sensitivity analysis: Even when the linear mixed model was performed without applying conditional mean imputation for missing data, the treatment-time interaction remained statistically significant in ADAS-Cog-14($F_{2,176}$=3.91, $\eta p2$ =0.031, P=.022). In the post-hoc analysis, no statistically significant differences were observed at baseline (estimates; 0.018, t_{232}=0.021, Bonferroni adjusted P=.984) and week 12(estimates; 1.53, t_{235}=1.69, Bonferroni adjusted P=.093). At week 24, the Bonferroni-adjusted P was .051, indicating a trend toward greater improvement in ADAS-Cog-14 scores in the ET-101 group compared to the control group (estimates; -2.13, t_{244}=-1.96). / Exploratory analysis: The results of the linear mixed model and post-hoc analysis for the subdomains of ADAS-Cog-14 were presented in Supplementary 3. Among the subdomains of ADAS-Cog-14, the treatment-time interaction in the memory domain was statistically significant ($F_{2,194}$=11.2, $\eta p2$ =0.07, P= <.001). In the post-hoc analysis, ET-101 showed a statistically significant improvement in the memory domain compared to the control group at week 24 (estimates; -2.50, t_{264}=-4.03, Bonferroni adjusted P=<.001).

The treatment-time interaction was also found to be statistically significant for the language domain ($F_{2,194}$=3.99, $\eta p2$ =0.027, P=.020). In the post-hoc analysis, ET-101 demonstrated a statistically significant improvement compared to the control group at week 24 (estimates=-0.807, t_{290}=-3.68, Bonferroni adjusted P=<.001). For the praxis subdomain, representing executive function, the treatment-time interaction was not statistically significant ($F_{2,194}$=0.497, $\eta p2$ =0.003, P=.666).

18-i) Subgroup analysis of comparing only users

A subgroup analysis of comparing only users is not uncommon in ehealth trials, but if done, it must be stressed that this is a self-selected sample and no longer an unbiased sample from a randomized trial (see 16-iii).

	1	2	3	4	5	
subitem not at all important	◉	○	○	○	○	essential

선택해제

Does your paper address subitem 18-i?

Copy and paste relevant sections from the manuscript (include quotes in quotation marks "like this" to indicate direct quotes from your manuscript), or elaborate on this item by providing additional information not in the ms, or briefly explain why the item is not applicable/relevant for your study

In this study, sensitivity analysis and exploratory analysis were conducted to compare the treatment group and the control group.

19) All important harms or unintended effects in each group
(for specific guidance see CONSORT for harms)

Does your paper address CONSORT subitem 19? *

Copy and paste relevant sections from the manuscript (include quotes in quotation marks "like this" to indicate direct quotes from your manuscript), or elaborate on this item by providing additional information not in the ms, or briefly explain why the item is not applicable/relevant for your study

In the ET-101 group, "six AEs occurred, while four AEs were reported in the control group, with no statistically significant difference in AE incidence between the groups (P=.525)". Among the AEs in the ET-101 group, "two were classified as SAEs due to prolonged hospitalization (Non-ST elevation myocardial infarction, herniated disc disease of lumbar spine)"; however, the incidence of SAEs also did not differ significantly between the two groups (P=.491). "All reported AEs were determined to be definitely not related to the ET-101".

19-i) Include privacy breaches, technical problems

Include privacy breaches, technical problems. This does not only include physical “harm” to participants, but also incidents such as perceived or real privacy breaches [1], technical problems, and other unexpected/unintended incidents. “Unintended effects” also includes unintended positive effects [2].

	1	2	3	4	5	
subitem not at all important	◉	○	○	○	○	essential

선택해제

Does your paper address subitem 19-i?

Copy and paste relevant sections from the manuscript (include quotes in quotation marks "like this" to indicate direct quotes from your manuscript), or elaborate on this item by providing additional information not in the ms, or briefly explain why the item is not applicable/relevant for your study

In this study, all participant-related data were anonymized and securely managed, ensuring that training results and other information were not disclosed externally.

19-ii) Include qualitative feedback from participants or observations from staff/researchers

Include qualitative feedback from participants or observations from staff/researchers, if available, on strengths and shortcomings of the application, especially if they point to unintended/unexpected effects or uses. This includes (if available) reasons for why people did or did not use the application as intended by the developers.

	1	2	3	4	5	
subitem not at all important	◉	○	○	○	○	essential

선택해제

Does your paper address subitem 19-ii?

Copy and paste relevant sections from the manuscript (include quotes in quotation marks "like this" to indicate direct quotes from your manuscript), or elaborate on this item by providing additional information not in the ms, or briefly explain why the item is not applicable/relevant for your study

In this study, dropout rates were calculated; however, no interviews were conducted to investigate the reasons for dropout or potential shortcomings of the application.

DISCUSSION

22) Interpretation consistent with results, balancing benefits and harms, and considering other relevant evidence

NPT: In addition, take into account the choice of the comparator, lack of or partial blinding, and unequal expertise of care providers or centers in each group

22-i) Restate study questions and summarize the answers suggested by the data, starting with primary outcomes and process outcomes (use)

Restate study questions and summarize the answers suggested by the data, starting with primary outcomes and process outcomes (use).

	1	2	3	4	5	
subitem not at all important	○	○	○	○	◉	essential

선택해제

Does your paper address subitem 22-i? *

Copy and paste relevant sections from the manuscript (include quotes in quotation marks "like this" to indicate direct quotes from your manuscript), or elaborate on this item by providing additional information not in the ms, or briefly explain why the item is not applicable/relevant for your study

This study investigated the efficacy and safety of ET-101 in patients with MCI, compared to a sham device control group. "We found that the ET-101 group showed significantly greater improvement in ADAS-Cog-14 score in 24 weeks than the control group". "Furthermore, no AEs related to the use of ET-101 were observed". Among the various digital cognitive trainings, to the best of our knowledge, this study is the first to investigate the effects of MMT in the form of mobile applications.

22-ii) Highlight unanswered new questions, suggest future research

Highlight unanswered new questions, suggest future research.

	1	2	3	4	5	
subitem not at all important	○	○	○	○	◉	essential

선택해제

Does your paper address subitem 22-ii?

Copy and paste relevant sections from the manuscript (include quotes in quotation marks "like this" to indicate direct quotes from your manuscript), or elaborate on this item by providing additional information not in the ms, or briefly explain why the item is not applicable/relevant for your study

Additional long-term studies are needed to investigate the effects of ET-101 on the rate of conversion from MCI to AD and to further assess its long-term efficacy. Given recent efforts to develop more sensitive scales for detecting changes in ADL, quality of life, and cognitive function in MCI, future studies using these updated tools will be essential for accurately evaluating the effects of ET-101.

20) Trial limitations, addressing sources of potential bias, imprecision, and, if relevant, multiplicity of analyses

20-i) Typical limitations in ehealth trials

Typical limitations in ehealth trials: Participants in ehealth trials are rarely blinded. Ehealth trials often look at a multiplicity of outcomes, increasing risk for a Type I error. Discuss biases due to non-use of the intervention/usability issues, biases through informed consent procedures, unexpected events.

	1	2	3	4	5	
subitem not at all important	○	○	●	○	○	essential

선택해제

Does your paper address subitem 20-i? *

Copy and paste relevant sections from the manuscript (include quotes in quotation marks "like this" to indicate direct quotes from your manuscript), or elaborate on this item by providing additional information not in the ms, or briefly explain why the item is not applicable/relevant for your study

In this study, the control group used a sham device that provided the same gamification, reminders, and cognitive tests as the treatment group, addressing potential blinding issues. Additionally, in the post hoc analysis, Bonferroni-adjusted p-values were reported to reduce Type I error.

21) Generalisability (external validity, applicability) of the trial findings

NPT: External validity of the trial findings according to the intervention, comparators, patients, and care providers or centers involved in the trial

21-i) Generalizability to other populations

Generalizability to other populations: In particular, discuss generalizability to a general Internet population, outside of a RCT setting, and general patient population, including applicability of the study results for other organizations

	1	2	3	4	5	
subitem not at all important	●	○	○	○	○	essential

선택해제

Does your paper address subitem 21-i?

Copy and paste relevant sections from the manuscript (include quotes in quotation marks "like this" to indicate direct quotes from your manuscript), or elaborate on this item by providing additional information not in the ms, or briefly explain why the item is not applicable/relevant for your study

This study enrolled only individuals who met the predefined and well-defined inclusion criteria for MCI to specifically evaluate the effects of digital therapy in this population. Therefore, the generalizability of the findings to other populations is limited.

21-ii) Discuss if there were elements in the RCT that would be different in a routine application setting

Discuss if there were elements in the RCT that would be different in a routine application setting (e.g., prompts/reminders, more human involvement, training sessions or other co-interventions) and what impact the omission of these elements could have on use, adoption, or outcomes if the intervention is applied outside of a RCT setting.

	1	2	3	4	5	
subitem not at all important	○	◉	○	○	○	essential

선택해제

Does your paper address subitem 21-ii?

Copy and paste relevant sections from the manuscript (include quotes in quotation marks "like this" to indicate direct quotes from your manuscript), or elaborate on this item by providing additional information not in the ms, or briefly explain why the item is not applicable/relevant for your study

In this study, the training process was conducted exclusively through a mobile app without any external intervention. The reminders provided during the training were generated by an in-app algorithm and were not influenced by the RCT setting.

OTHER INFORMATION

23) Registration number and name of trial registry

Does your paper address CONSORT subitem 23? *

Copy and paste relevant sections from the manuscript (include quotes in quotation marks "like this" to indicate direct quotes from your manuscript), or elaborate on this item by providing additional information not in the ms, or briefly explain why the item is not applicable/relevant for your study

ClinicalTrials.gov NCT05938426

24) Where the full trial protocol can be accessed, if available

Does your paper address CONSORT subitem 24? *

Cite a Multimedia Appendix, other reference, or copy and paste relevant sections from the manuscript (include quotes in quotation marks "like this" to indicate direct quotes from your manuscript), or elaborate on this item by providing additional information not in the ms, or briefly explain why the item is not applicable/relevant for your study

This information can be found in the Study Design and Participants section of the Methods. Additionally, the prespecified procedures for the clinical trial can be accessed on ClinicalTrials.gov (NCT05938426).

25) Sources of funding and other support (such as supply of drugs), role of funders

Does your paper address CONSORT subitem 25? *

Copy and paste relevant sections from the manuscript (include quotes in quotation marks "like this" to indicate direct quotes from your manuscript), or elaborate on this item by providing additional information not in the ms, or briefly explain why the item is not applicable/relevant for your study

This research was supported by the Ministry of Trade, Industry and Energy, Korea, under the Regional Innovation Cluster Development Program(R&D, P0025292) supervised by the Korea Institute for Advancement of Technology.

X27) Conflicts of Interest (not a CONSORT item)

X27-i) State the relation of the study team towards the system being evaluated

In addition to the usual declaration of interests (financial or otherwise), also state the relation of the study team towards the system being evaluated, i.e., state if the authors/evaluators are distinct from or identical with the developers/sponsors of the intervention.

	1	2	3	4	5	
subitem not at all important	○	○	○	◉	○	essential

선택해제

Does your paper address subitem X27-i?

Copy and paste relevant sections from the manuscript (include quotes in quotation marks "like this" to indicate direct quotes from your manuscript), or elaborate on this item by providing additional information not in the ms, or briefly explain why the item is not applicable/relevant for your study

In this study, authors with no competing interests were explicitly identified. Additionally, any authors who were employed by or received funding from Emocog Inc., the developer of ET-101, were disclosed in the conflict of interest statement.

About the CONSORT EHEALTH checklist

As a result of using this checklist, did you make changes in your manuscript? *

○ yes, major changes

○ yes, minor changes

◉ no

What were the most important changes you made as a result of using this checklist?

내 답변

How much time did you spend on going through the checklist INCLUDING making changes in your manuscript *

I spent 1 hours to go through the checklist

As a result of using this checklist, do you think your manuscript has improved? *

- (●) yes
- ( ) no
- ( ) 기타:

Would you like to become involved in the CONSORT EHEALTH group?

This would involve for example becoming involved in participating in a workshop and writing an "Explanation and Elaboration" document

- ( ) yes
- (●) no
- ( ) 기타:

선택해제

Any other comments or questions on CONSORT EHEALTH

No additional comments.

! Your answer must have a minimum of 25 characters.

STOP - Save this form as PDF before you click submit

To generate a record that you filled in this form, we recommend to generate a PDF of this page (on a Mac, simply select "print" and then select "print as PDF") before you submit it.

When you submit your (revised) paper to JMIR, please upload the PDF as supplementary file.

Don't worry if some text in the textboxes is cut off, as we still have the complete information in our database. Thank you!

Final step: Click submit !

Click submit so we have your answers in our database!

제출 양식 지우기

Google Forms를 통해 비밀번호를 제출하지 마세요.

이 설문지는 도메인의 외부에서 만들었습니다. - 서비스 약관 - 개인정보처리방침

Does this form look suspicious? 보고서

Google 설문지
